# Transcription Factors OVOL1 and OVOL2 Induce the Mesenchymal to Epithelial Transition in Human Cancer

**DOI:** 10.1371/journal.pone.0076773

**Published:** 2013-10-04

**Authors:** Hernan Roca, James Hernandez, Savannah Weidner, Richard C. McEachin, David Fuller, Sudha Sud, Taibriana Schumann, John E. Wilkinson, Alexander Zaslavsky, Hangwen Li, Christopher A. Maher, Stephanie Daignault-Newton, Patrick N. Healy, Kenneth J. Pienta

**Affiliations:** 1 Department of Urology, University of Michigan School of Medicine, Ann Arbor, Michigan, United States of America; 2 Department of Internal Medicine, University of Michigan School of Medicine, Ann Arbor, Michigan, United States of America; 3 Department of Pathology, University of Michigan School of Medicine, Ann Arbor, Michigan, United States of America; 4 Department of Computational Medicine and Bioinformatics, University of Michigan School of Medicine, Ann Arbor, Michigan, United States of America; 5 Division of Biostatistics, Comprehensive Cancer Center, University of Michigan School of Medicine, Ann Arbor, Michigan, United States of America; 6 The Genome Institute, Division of Oncology, Department of Medicine, Washington University School of Medicine, St. Louis, Missouri, United States of America; The University of Kansas Medical center, United States of America

## Abstract

Cell plasticity regulated by the balance between the mesenchymal to epithelial transition (MET) and the opposite program, EMT, is critical in the metastatic cascade. Several transcription factors (TFs) are known to regulate EMT, though the mechanisms of MET remain unclear. We demonstrate a novel function of two TFs, OVOL1 and OVOL2, as critical inducers of MET in human cancers. Our findings indicate that the OVOL-TFs control MET through a regulatory feedback loop with EMT-inducing TF ZEB1, and the regulation of mRNA splicing by inducing Epithelial Splicing Regulatory Protein 1 (ESRP1). Using mouse prostate tumor models we show that expression of OVOL-TFs in mesenchymal prostate cancer cells attenuates their metastatic potential. The role of OVOL-TFs as inducers of MET is further supported by expression analyses in 917 cancer cell lines, suggesting their role as crucial regulators of epithelial-mesenchymal cell plasticity in cancer.

## Introduction

More than 90% of cancer-related deaths result from metastasis [1]. Consequently, we must further our understanding of the mechanisms driving cancer progression and metastasis. Epithelial to mesenchymal transition (EMT), and the converse process of MET, are crucial programs involved in wound healing and early organ development [2]. EMT and MET may also play critical roles in both cancer progression and establishment of metastatic colonies [3]. When cancer cells undergo EMT, they acquire the ability to dissociate from the primary tumor and enter circulation. These circulating cells are then capable of disseminating and undergoing MET, resulting in metastatic tumors. Understanding this phenotypic plasticity is one key to hindering cancer progression [4].

EMT is linked to alterations in gene expression and morphology and is associated with downregulation of cell adhesion proteins, such as E-cadherin (E-cad). This process allows cancer cells to dissociate from their neighbors while increasing their motility [5]. It has been proposed that EMT is regulated by reciprocal feedback loops between ZEB1/ZEB2 TFs and members of the MicroRNA miR-200 family [6,7]. Specifically, ZEB1 can induce EMT by repressing epithelial proteins and by downregulating its own miR-200 repressors. At the same time, miR-200s repress ZEB1 as well as stem cell factors and epigenetic regulators involved in EMT. It is thought that these reciprocal feedback loops are responsible, in part, for the phenotypic plasticity exhibited in cancer and metastasis [4].

Other proteins may serve as regulators/inducers of EMT or potential biomarkers for mesenchymal cells. Induction of EMT has been associated with TGFβ expression [8]. In part, this occurs via upregulation of ZEB1/2, which, in turn, inhibits epithelial splicing regulatory proteins (ESRP) [9]. Downregulation of ESRP, and the resulting repression of alternative splicing, has been shown to be crucial in EMT. For example, in breast cancer, repression of ESRP results in a switch in CD44 isoform expression that is critical in the induction of EMT and cancer progression [10].

Despite our growing knowledge regarding EMT, the mechanisms mediating MET are far less understood. Using two models of prostate and breast cancer mesenchymal cells, we discovered that TFs OVOL1 (OVO-like 1, Entrez Gene ID 5017) and OVOL2 (Gene ID 58495) are associated with MET in our models, as well as in other cancers. OVOLs are zinc-finger TFs that act as regulators of embryogenesis [11–13]. We first analyzed how OVOLs control expression of EMT-inducing TFs and ESRPs. We then studied how MET, induced by the OVOL-TFs, correlates with key factors of epithelial cell development in 917 cancer cell lines that conform to the Human Cancer Cell Encyclopedia [14], and investigated the implications of MET in the regulation of cancer cell invasion and metastasis.

## Results

### Stable mesenchymal cells isolated from stable epithelial prostate cancer cells co-cultured with macrophages

**Figure 1:**
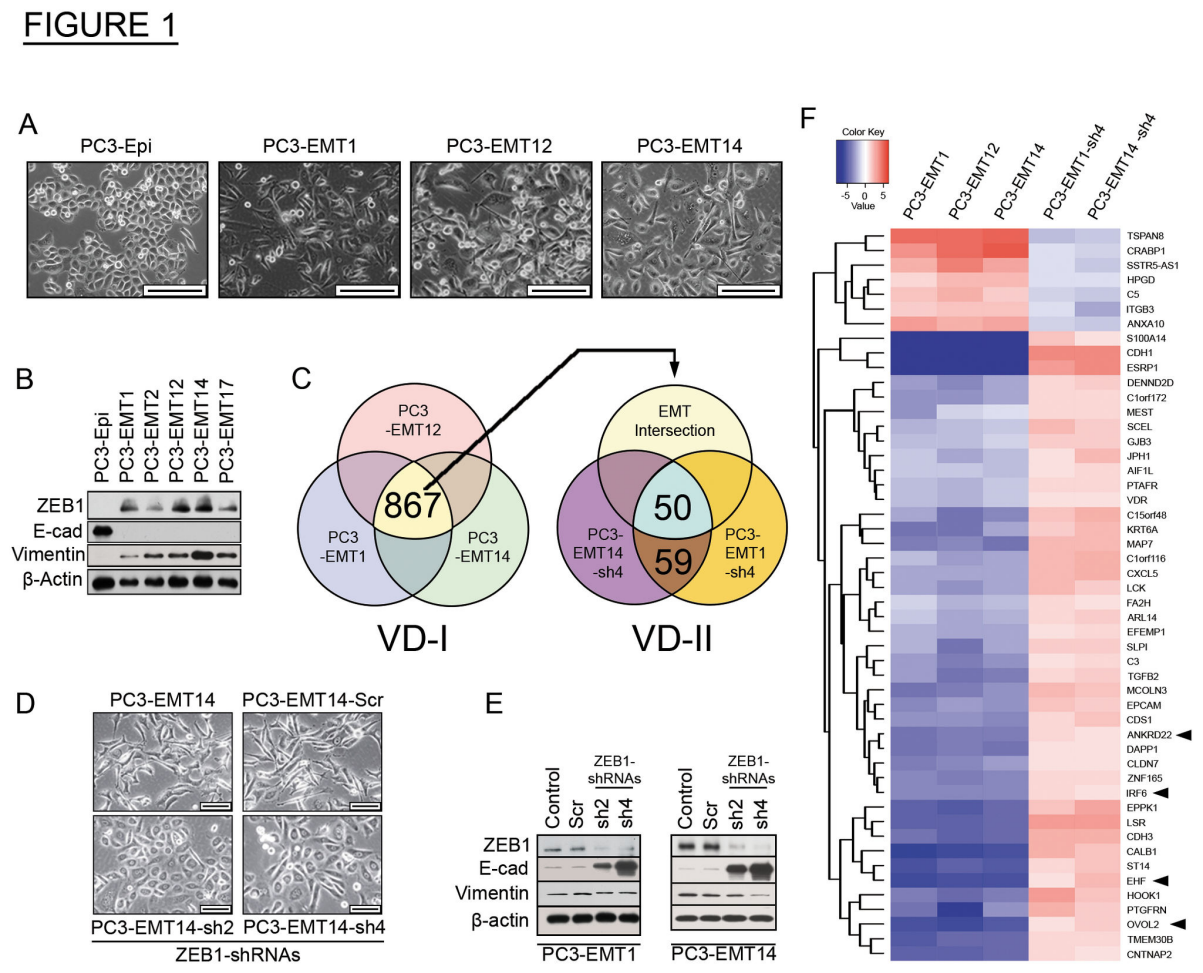
Mesenchymal cancer cell populations isolated from co-cultures of epithelial prostate cancer cells and human macrophages. (A) Bright-phase microscopy: morphology changes in the mesenchymal cells PC3-EMT1, PC3-EMT12 and PC3-EMT14 as compared to the epithelial PC3-Epi prostate cancer cells. Scale bars are 200 µm. (B) Immunoblot: Expression of EMT markers in mesenchymal cancer cell lines PC3-EMT1, -EMT2, -EMT12, -EMT14 and -EMT17 compared to epithelial PC3-Epi. (C) Microarray: Gene expression analyses comparing mesenchymal to epithelial cancer cell lines. Venn diagram-I (VD-I) depicts a common EMT-associated signature expressed in the mesenchymal cancer lines PC3-EMT1, -EMT12 and -EMT14 compared to epithelial PC3-Epi. VD-II – Gene signature from VD-I intersected with the signature of ZEB1 silenced cells PC3-EMT1 and -EMT14 using the shRNA-sh4, relative to scramble (Scr) control shRNA. (D) Bright-phase microscopy: PC3-EMT14 cells transfected with ZEB1-shRNAs: sh2, sh4, or Scr. Scale bars are 100 µm. (E) Immunoblot: Expression of EMT markers in PC3-EMT1 and -EMT14 transfected with ZEB1-shRNAs compared to Scr or non-transfected controls. (F) Heat map: 50 genes signature identified in VD-II (panel C). Upregulated genes are in red, downregulated in blue. Fold changes in mesenchymal cancer cells are relative to epithelial PC3-Epi, and in sh4-transfected cells are relative to the Scr control. The immunoblots shown are representative of two independent experiments with similar results. See also Figure S1 and Table S1.

To study the mechanisms of EMT in prostate cancer metastasis we first generated an epithelial model cell line derived from luciferase positive prostate cancer epithelial PC3 cells. We isolated a subpopulation of cells expressing luciferase that showed a stable epithelial phenotype in culture and designated them PC3-Epi. These cells were selected based on two criteria: bioluminescent intensity and epithelial cell morphology. Consistent with the epithelial state, PC3-Epi cells maintained high E-cad, low Vimentin, and undetectable expression of the EMT-activator ZEB1 (Figure 1A and 1B).

We next developed a mesenchymal model from PC3-Epi cells, through interactions with macrophages. We hypothesized that this would be possible because macrophages represent one of the major inflammatory cell-infiltrates in the tumor microenvironment and they have been implicated in metastasis [15,16]. Co-culture of IL4-treated CD14+ monocytes (M2-macrophages) with PC3-Epi for four days induced strong morphologic alterations consistent with EMT, with concomitant decline in the expression of E-cad and increase in Vimentin (Figures S1A and S1B). From these cells we isolated mesenchymal sub-populations designated as PC3-EMT1, PC3-EMT2, PC3-EMT12, PC3-EMT14 and PC3-EMT17. These mesenchymal cells demonstrated a striking phenotypic alteration and expression of ZEB1, which was stably maintained in culture (Figure 1A and 1B). Compared to the parental epithelial PC3-Epi the mesenchymal cells expressed lower levels of the epithelial cell adhesion molecule EpCAM; however, still about 45% of the cells expressed EpCAM in the cell surface as demonstrated by flow cytometry (Figure S1C).

To assess gene expression changes related to EMT we performed three microarray analyses, comparing PC3-EMT1, PC3-EMT12 and PC3-EMT14 to PC3-Epi. We identified a signature of 867 genes that show changes in expression (by 2 fold or more) in all three comparisons, (VD-I, Figure 1C). We used Ingenuity Pathway Analysis (Ingenuity Systems, www.ingenuity.com) to ascertain the most significantly enriched molecular functions associated with this signature: ‘cellular movement’, ‘cellular growth and proliferation’, ‘cell-to-cell signaling and interaction’, and ‘cell death’ are all consistent with EMT (Table S1). ZEB1, which was upregulated in all three comparisons, has been widely implicated in EMT and maintenance of the mesenchymal state [2,7]. Other EMT-inducing transcription factors like Snail, Slug or Twist1, frequently implicated in tumorigenesis and metastasis [2], were not identified among the common EMT signature of 867 genes described above. Therefore to understand the role of ZEB1 in the EMT cell program, we transfected two mesenchymal lines (PC3-EMT1 and PC3-EMT14) with one of two lentiviral ZEB1-shRNAs vectors: sh2 and sh4, plus the scrambled control (Scr). Consistent with ZEB1’s role in EMT and maintenance of the mesenchymal state, silencing of ZEB1 induced MET in PC3-EMT1 and PC3-EMT14, with corresponding changes in cell morphology (Figure 1D). Furthermore, sh2 and sh4 induced a striking decline in ZEB1, corresponding to upregulation of E-cad (Figure 1E). These experiments strongly suggest an essential role of ZEB1 in the EMT state of these cells. When the set of differentially expressed genes in sh4-transfected cells was matched to the EMT signature (VD-I), we found expression changes in 50 genes that correlate with MET transformation (VD-II, Figure 1C). This signature showed opposite regulation when the cells were transfected with ZEB1-shRNA sh4, relative to the scrambled control (Figure 1F). This approach also identified a set of 4 TFs significantly downregulated in the mesenchymal cells and upregulated by ZEB1-shRNA: IRF6, ANKRD22, EHF and OVOL2.

### OVOL1 and OVOL2 regulate MET in the prostate cancer model

**Figure 2:**
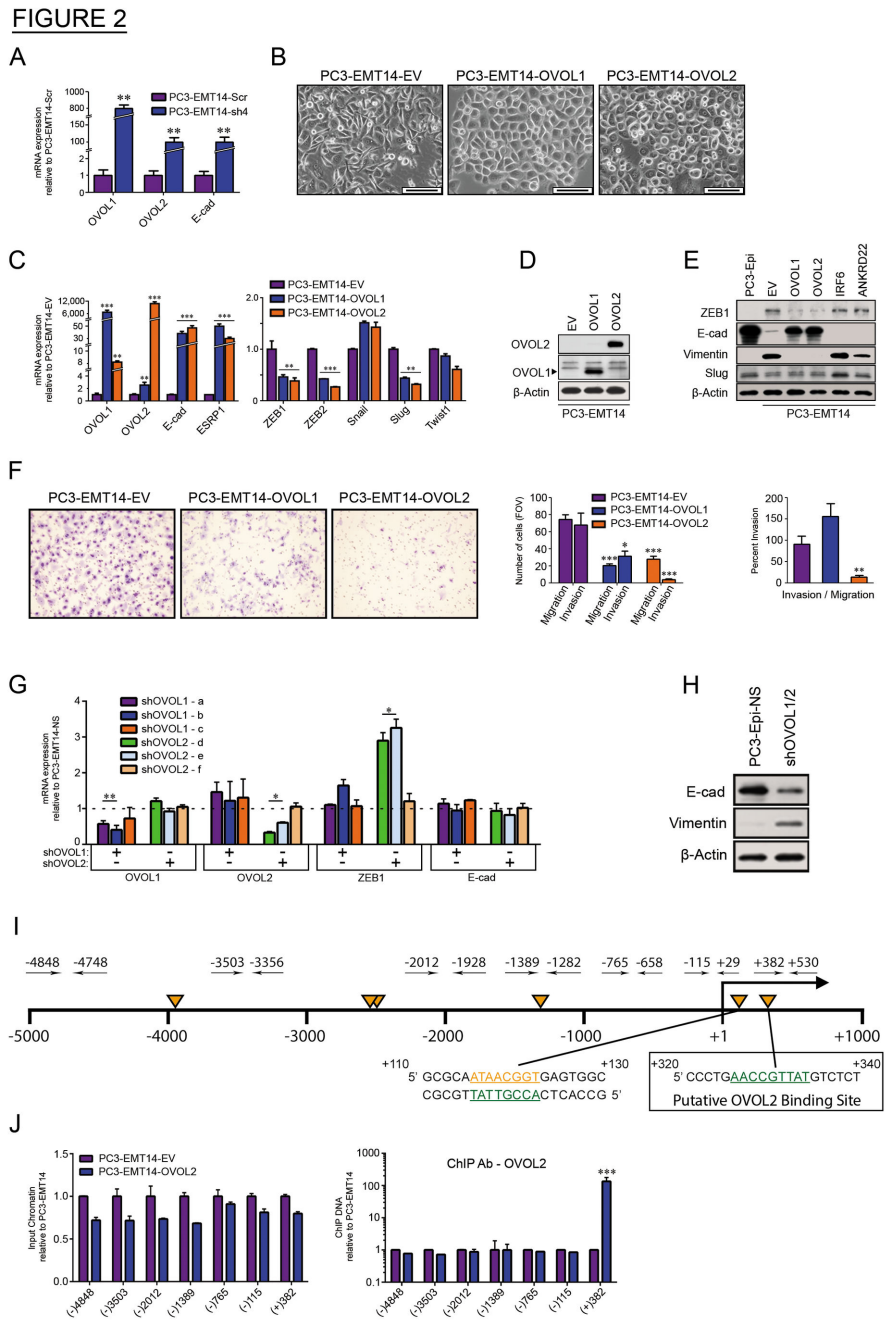
OVOL1 and OVOL2 induce MET in mesenchymal prostate cancer cells. (A) qPCR: mRNA expression in PC3-EMT14-sh4 relative to PC3-EMT14-Scr. (B) Bright-phase microscopy: PC3-EMT14 cells expressing OVOL1 and OVOL2 exhibit an epithelial phenotype as compared to the parental PC3-EMT14. Scale bars are 100 µm. (C) qPCR: Expression of the epithelial cell markers E-cad and ESRP1, and the EMT-inducing TFs ZEB1, ZEB2, Slug, Snail, and Twist1 in PC3-EMT14 cells expressing OVOL1 and OVOL2 relative to the empty-vector (EV) control. (D) Immunoblot: OVOL1 and OVOL2 protein expression in transfected cells was confirmed by using OVOL1 or V5-specific antibodies, respectively. (E) Immunoblot: Expression of epithelial marker E-cad and mesenchymal markers ZEB1 and Vimentin. Changes in Slug expression were minimal and did not correlate with MET. PC3-EMT14 cells expressing IRF6 or ANKRD22, EV and epithelial PC3-Epi are controls. (F) Invasion/migration assay: Representative images and graphs of cancer cell invasion using a Boyden chamber assay. Bar graphs of OVOL1 and OVOL2 expressing PC3-EMT14 cells relative EV. Percent invasion represents the ratio invading/migrating cells. The graph is representative of one out of three independent experiments. (G) qPCR: Expression of PC3-Epi cells transfected with OVOL1-shRNAs (a, b, c) or OVOL2-shRNAs (d, e, f), relative to the non-silencing control PC3-Epi-NS (represented by the dashed line). (H) Immunoblot: PC3-Epi cells transfected with both OVOL1 and OVOL2 shRNAs (sh-OVOL1/2) demonstrate a decrease in E-cad and increase in Vimentin, which suggests a partial EMT transformation. (I) Schematic: The ZEB1 promoter with potential OVOL2 binding sites (orange triangles) according to the general consensus: 5’-A(A/T) (A/T) (C/A) (T/C)GTTA(T/A). Designed TaqMan primer-pairs are shown as black arrows. Numeration is relative to the transcriptional start site (+1). The sequence of a putative OVOL2 binding site that corresponds to the ChIP DNA in the OVOL2 expressing cells is at +320. (J) ChIP qPCR: (Left) shows input chromatin of PC3-EMT14-OVOL2 relative to EV, and demonstrates that similar amounts of DNA were used (Right). depicts the ChIP DNA using OVOL2 antibody. Primers are named after their forward primer (see panel I). Results were normalized to input controls. The graph depicts one representative experiment out of three with similar results. qPCR results were normalized to β –actin. Graphs show mean +/- sem; p-values are represented as * p < 0.05; ** p < 0.01; *** p < 0.001. The qPCRs and immunoblots are representative of two independent experiments with similar results. See also Figure S2.

Given the observed changes in expression of IRF6, ANKRD22, EHF and OVOL2 in EMT, as well as the observed feedback between ZEB1 and these TFs, we speculated that the stable EMT characteristics maintained during propagation of PC3-EMT1 and PC3-EMT14 cells are a result of a regulatory feedback loop. Equally, since these genes were downregulated in EMT, overexpressing them may induce MET. We assessed the role of each of the 4 upregulated TFs individually by transducing PC3-EMT14 cells with lentiviral overexpressing constructs. The expression of these 4 TFs in the EMT cells demonstrated that OVOL2 appeared to regulate MET, as observed by a change in cell morphology. Given the strong parallels between OVOL2 and OVOL1, as well as OVOL2’s apparent role in MET, we further considered whether OVOL1 may have a role in MET. Though OVOL1 did not meet the 2-fold threshold to be included in the microarray gene signature of ZEB1 knockdown-cells, the qPCR analysis showed that it is highly upregulated in the epithelial PC3-Epi cells and following ZEB1 silencing compared to the mesenchymal PC3-EMT14 (Figure 2A and Figure S2A). Therefore, we also examined the expression of OVOL1 in the mesenchymal PC3-EMT14 cells. Overexpression of both OVOL1 and OVOL2 induced a marked transformation of cell morphology and a parallel increase in E-cad expression (Figure 2B and 2C). Furthermore, both OVOLs increased expression of ESRP1 (Figure 2C, left panel) [17]. By qPCR we then analyzed the changes in the expression of several EMT-inducing transcription factors: ZEB1, ZEB2, Snail, Slug and Twist1 (Figure 2C, right panel). Among these factors ZEB1, ZEB2 and Slug revealed a significant decrease in expression, which correlates with MET. We confirmed that expression changes seen in OVOL1 and OVOL2 correspond to changes in protein levels by Western blot (Figure 2D). We then compared cell lysates from OVOL1 and OVOL2-overexpressing cells to PC3-Epi, PC3-EMT14-EV, and cells overexpressing the TFs IRF6 and ANKRD22, also regulated by ZEB1 (Figure 1F). Rescue of E-cad and loss of Vimentin were induced in both OVOL-overexpressing cells, while no significant change was observed in the cells expressing other TFs (IRF6 and ANKRD22) (Figure 2E). Although qPCR reflected a significant change in Slug expression, the Western did not show a change at the protein level in the OVOL overexpressing cells, suggesting that translational regulation might play a role in Slug levels. Of the five TFs tested, these results indicate that OVOL1 and OVOL2 are key inducers of MET in these cells.

To understand the implication of OVOL-mediated MET in the invasive potential of mesenchymal PC3-EMT14 cells, we used Boyden chamber assay on cells overexpressing OVOL1/2. Both OVOLs significantly affected the invasion capacity of PC3-EMT14. In the case of OVOL1, we observed reduced cell migration, which affected the ratio of invasion vs. migration (invasion index) (Figure 2F). OVOL2 overexpressing cells demonstrated a significant decline in both invasion and migration, with a highly reduced invasion index. These results support the role of OVOL1 and OVOL2 in decreasing migration and invasion, as expected for MET reprogrammed cells.

We next used shRNAs for OVOL1 (shOVOL1-a, -b, -c) and OVOL2 (shOVOL2-d, -e, -f), to reduce their expression in epithelial PC3-Epi cells (Figure 2G). None of the shRNAs affected E-cad expression. However, in cells with decreased OVOL2 expression, we observed a significant upregulation (up to 3-fold) of ZEB1 mRNA. These results suggest that a 3-fold change in ZEB1 expression is not sufficient to induce EMT in PC3-Epi cells. Moreover, as observed by qPCR, the expression of ZEB1 is over 20 fold higher in PC3-EMT14 compared to PC3-Epi cells (Figure S2A). Furthermore given the similar function of OVOL1 and OVOL2 in the induction of MET it is tempting to speculate that they could substitute for one another. This may explain why a knockdown of only one of the OVOL is not sufficient to induce a significant change in E-cad. To test this possibility we performed a double knockdown with shRNAs for each of the OVOL-TFs. Although no selection of transfected cells was possible (both shRNA constructs expressed GFP), a total population of cells transfected with shOVOL1-a and shOVOL2-d (shOVOL1/2-a/d) demonstrated a significant upregulation of vimentin and a partial decrease in E-cad compared to the control PC3-Epi-NS cells (Figure 2H). Overall, these findings suggest a critical role of both OVOLs in maintaining the epithelial state of these cells and therefore, a double knockdown would be required to induce and maintain the mesenchymal state. Consequently, a greater knockdown of OVOL1 and OVOL2 would induce a more complete EMT transformation in cancer cells.

Other experiments in prostate cancer cells induced to undergo EMT led to similar findings concerning the regulation of the OVOL and ZEB1 TFs. For example, the transmembrane protein Tetraspanin-8 (TSPAN8) was identified in the signature shown in Figure 1F as a gene upregulated in EMT cells and downregulated by ZEB1-shRNA. Overexpression of TSPAN8 in the epithelial PC3-Epi and DU145 cells led to partial EMT transformation characterized by the upregulation of ZEB1 and the downregulation of the OVOL TFs in both cell lines (Figure S2B-E).

A transcriptional repressor function of OVOL2 was previously shown in keratinocytes, where OVOL2 repressed c-Myc and Notch1 [18]. Since ZEB1 expression significantly increased in cells with decreased OVOL2 (Figure 2G), we tested if OVOL2 directly interacts with the ZEB1 promoter. We performed chromatin immunoprecipitation (ChIP) targeting the ZEB1 promoter in PC3-EMT14-OVOL2 and control cells with two antibodies, anti-OVOL2 and anti-V5-tag. We designed 7 TaqMan primers and probes spanning the promoter from -4848 to +530 bp (Figure 2I) and found several potential OVOL2 binding sites: 5’-A(A/T) (A/T) (C/A) (T/C)GTTA(T/A) [18]. With both antibodies, we observed over 50-fold enrichment for the same primer/probe-position (+382/+530) when comparing OVOL2-expressing and control cells (Figure 2J and Figure S2B). In contrast, input chromatin of both cell-types showed similar amplification with all primer-pairs. These results indicate specific binding of OVOL2 to the ZEB1 promoter at the +325 region, where one OVOL2 consensus site was identified (Figure 2I). In addition, since no specific chromatin fragment was amplified with -115/+29 primers, it is unlikely that OVOL2 binds to the +115 site, another potential site in the proximal region (Figure 2I). Fragment +123 -(-115) = 238 bp is significantly smaller than +530 – (+115) = 415 bp; but about the same size as + 530 – (+325) = + 205 bp. Therefore, a binding to the +115 site would imply a specific pull-down of a chromatin fragment that should be detected with the -115/+29 primers. Together with the shRNA and expression data, these findings suggest that OVOL2 acts as a direct transcriptional repressor of ZEB1.

### Mesenchymal cells form mesenchymal tumors in vivo

**Figure 3:**
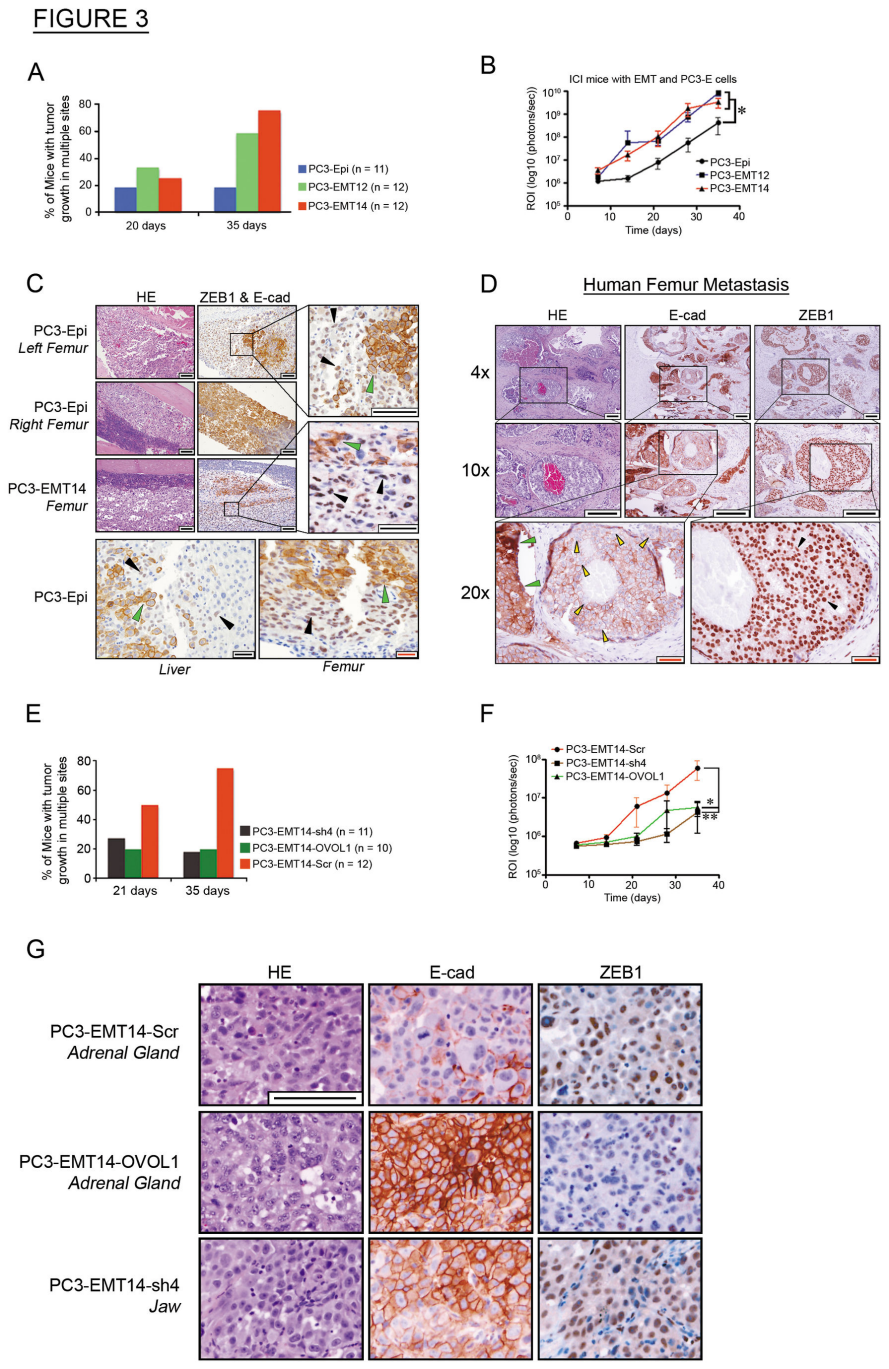
Mesenchymal cancer cells show increased metastasis while not requiring MET for solid tumor formation. (A) Metastasis: The percentage of ICI-inoculated mice with multiple luciferase signals at 21 and 35 days. (B) Tumor burden: Mice received ICI and were imaged weekly for 35 days. Luciferase expression is represented as regions of interest (ROI-photons/s). (C) IHC: Simultaneous ZEB1 and E-cad staining of metastases detected in the femur and liver of PC3-Epi or PC3-EMT14 injected mice. Note the mesenchymal (ZEB1^high^ / E-cad^low^) (black arrows) and epithelial (ZEB1^low^ / E-cad^high^) (green arrows) cancer cells, depicting the EMT/MET plasticity in subpopulations of PC3-Epi and PC3-EMT14. Scale bars are 100 µm (black) and 20 µm (red). (D) IHC: ZEB1 or E-cad staining of metastasis from the femur of a patient with advanced prostate cancer. Note the mesenchymal-like (ZEB1^high^ (black arrows)/ E-cad^low^ (yellow arrows)) and epithelial (ZEB1^low^ / E-cad^high^ (green arrows)) cancer cells. Scale bars shown represent 100 µm (black bar) and 50 µm (red bar). (E) Metastasis: The percentage of ICI-inoculated mice with multiple luciferase signals at 21 and 35 days. (F) Tumor burden: Mice received ICI and were imaged weekly for 35 days. Luciferase expression is depicted as regions of interest (ROI-photons/s). (G) IHC: ZEB1 or E-cad staining of metastases in ICI-mice. Note the higher E-cad and lower ZEB1 expression in the metastatic cells expressing OVOL1 or ZEB1-shRNA (sh4). Scale bar represents 100 µm. Graphs show mean +/- sem; p-values were calculated and represented as * p < 0.05; ** p < 0.01. All IHC images are representative of one out of three sections showing similar results. See also Figure S3.

To determine the tumorigenic potential of PC3-EMT12 and PC3-EMT14, we subcutaneously inoculated immunocompromised NSG mice. Both mesenchymal cancer cell lines formed predominantly mesenchymal-type tumors characterized by low E-cad and high ZEB1, while PC3-Epi tumors showed high E-cad and low ZEB1 expression (Figure S3A). We saw no significant differences in tumor growth rates between the groups (Figure S3B). To assess the metastatic potential of the mesenchymal cells, we used the intracardiac injection (ICI) mouse model of cancer metastasis [19]. We observed more mice with metastasis in multiple sites in the groups injected with PC3-EMT12 or PC3-EMT14 cells (Figure 3A). They also showed a higher overall tumor burden (about one order of magnitude) and decreased mouse survival, in comparison to mice injected with parental PC3-Epi cells (Figure 3B and Figure S3C). Simultaneous staining with E-cad and ZEB1 antibodies demonstrated that metastatic PC3-EMT12 and PC3-EMT14 tumors displayed typical mesenchymal characteristics (low E-cad and high ZEB1), while metastatic tumors from PC3-Epi cells displayed predominantly epithelial characteristics (high E-cad and low ZEB1) (Figure 3C and Figure S3D-E). Our findings suggest that mesenchymal cells (PC3-EMT12 and PC3-EMT14) form metastases that maintain the mesenchymal phenotype. Notably, some metastatic tumors from PC3-Epi cells exhibited both mesenchymal and epithelial characteristics, though a majority of the PC3-Epi tumors demonstrated the epithelial phenotype (Figure 3C). Figure 3C shows a mouse injected with PC3-Epi cells, where a tumor in the left femur was predominantly mesenchymal type, while the tumor in the right displayed a highly epithelial phenotype. Importantly, we found similar mesenchymal-epithelial cell plasticity in a metastatic tumor isolated from the femur of a patient with advanced prostate cancer. IHC staining of tumor sections with E-cad and ZEB1 antibodies showed regions with both highly epithelial (high E-cad, low ZEB1) and with mesenchymal characteristics (low E-cad and high ZEB1) (Figure 3D).

According to the results shown in Figure 2B-E, the effect of OVOL1 overexpression is similar to OVOL2. They both induce MET and ZEB1 downregulation. In addition ZEB1-shRNA induces the expression of both OVOL1 and OVOL2 and MET in the mesenchymal PC3-EMT14 cells (Figure 2A). To elucidate the role of OVOL TFs in the metastatic potential of the cells, we inoculated mice via ICI with epithelial cells overexpressing OVOL1 (PC3-EMT14-OVOL1), ZEB1-shRNA (PC3-EMT14-sh4) or the scrambled control (PC3-EMT14-Scr). We assessed tumor progression by bioluminescence and observed that induced MET in PC3-EMT14 cells significantly reduced metastatic potential. Both ZEB1-shRNA and OVOL1 expression reduced the number of mice with tumor metastasis in multiple sites (Figure 3E). Furthermore, the overall tumor burden declined significantly when compared to mice injected with PC3-EMT14-Scr control cells (Figure 3F). IHC staining of metastatic tumors with E-cad and ZEB1 revealed that MET had occurred in tumor cells that express OVOL1 or ZEB1-shRNA, as indicated by positive E-cad and lowered ZEB1 expression in contrast to the mesenchymal control cells (Figure 3G).

### OVOL-induced MET reduces the metastatic potential of mesenchymal cancer cells

**Figure 4:**
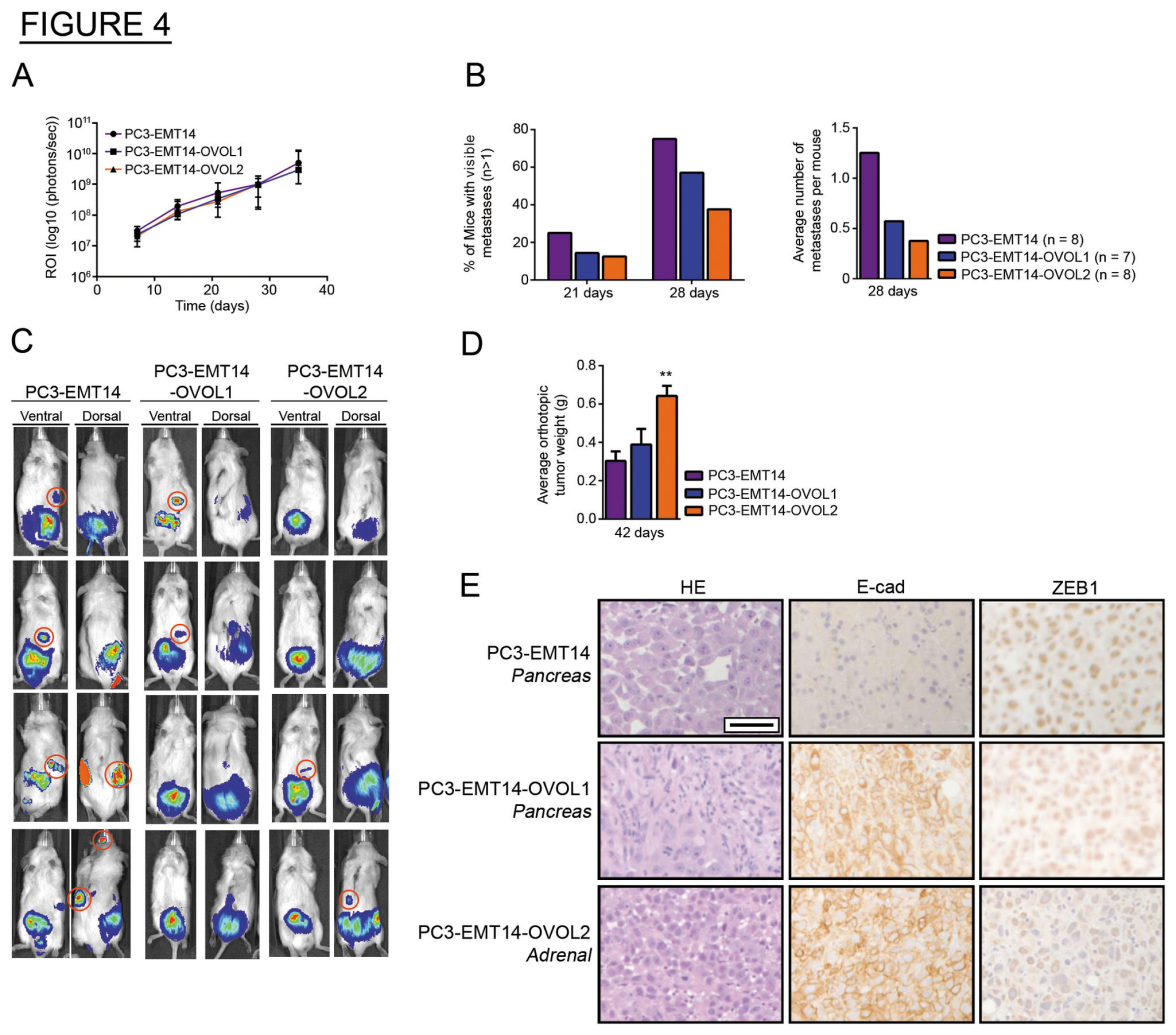
OVOL-induced MET reduces the metastatic potential of mesenchymal PC3-EMT14 in vivo. (A) Tumor burden: Luciferase expression is depicted as regions of interest (ROI-photons/s) in mice with orthotopic injections. (B) Metastasis: (Left) the percentage of orthotopically inoculated mice with multiple luciferase signals at 21 and 28 days (Right). depicts the total number of metastases per group divided by the number of mice (n) per group at 28 days. (C) Imaging: Representative images of luciferase expression in mice 28 days after receiving orthotopic injections. Metastases for each group are circled in red in either the ventral or dorsal images and excluding those suspected to correspond to the same tumor. (D) Tumor Weight: The average weights of orthotopic (prostate) tumors resected at 42 days (n = 4). (E) IHC: E-cad, and ZEB1 staining of metastatic tumors in mice that received orthotopic injections from each group inoculated with OVOL expressing cells or control PC3-EMT14. Note the higher E-cad and lower ZEB1 expression in OVOL-expressing cancer cells. Scale bar represents 100 µm. The IHC shows a representative staining of one out of three sections with similar results. Graphs show mean +/- sem; p-values were calculated and represented as ** p < 0.01. See also Figure S4.

To further investigate the metastatic potential of the OVOL-expressing cells, we used an orthotopic mouse model of prostate cancer [20]. In bioluminescence analysis, we saw a similar total tumor burden (ROIs) in all groups (Figure 4A). Twenty-eight days after inoculation, the number of mice with metastases was significantly lower in the group injected with OVOL-expressing cells (Figure 4B and 4C). We resected primary tumors 42 days after inoculation, recorded their weights, and analyzed tumor sections by IHC. Orthotopic prostate tumor weights were significantly higher in mice inoculated with PC3-EMT14-OVOL2, while mice injected with control PC3-EMT14 cells showed the lowest weights (Figure 4D). The observed decrease in metastatic potential for OVOL-expressing cells in our model correlates with a previous report showing that mice with larger orthotopic prostate tumors had a reduced metastatic burden [20].

IHC staining of the tumors revealed that PC3-EMT14 tumors (orthotopic and metastases) are predominantly mesenchymal and are mostly negative for E-cad expression and positive for ZEB1 (Figure 4E and Figure S4A). These mesenchymal cells can proliferate and form metastasis. This was further demonstrated by Ki67 positive staining of E-cad negative cells in metastatic tumor sections of PC3-EMT14 mice (Figure S4B). Although we cannot exclude the possibility of transient MET followed by EMT, these findings suggest that mesenchymal cells do not need to undergo MET to colonize the tumor. In contrast, the OVOL-expressing cells formed orthotopic tumors and metastases with strong epithelial phenotype, characterized by high expression of E-cad and lowered ZEB1; which strongly suggests that the OVOL-TFs induce and stabilize MET in these cells (Figure 4E and Figure S4A).

### OVOL TFs orchestrate a transcriptional and splicing-regulatory program that induces MET in human cancer cells

**Figure 5:**
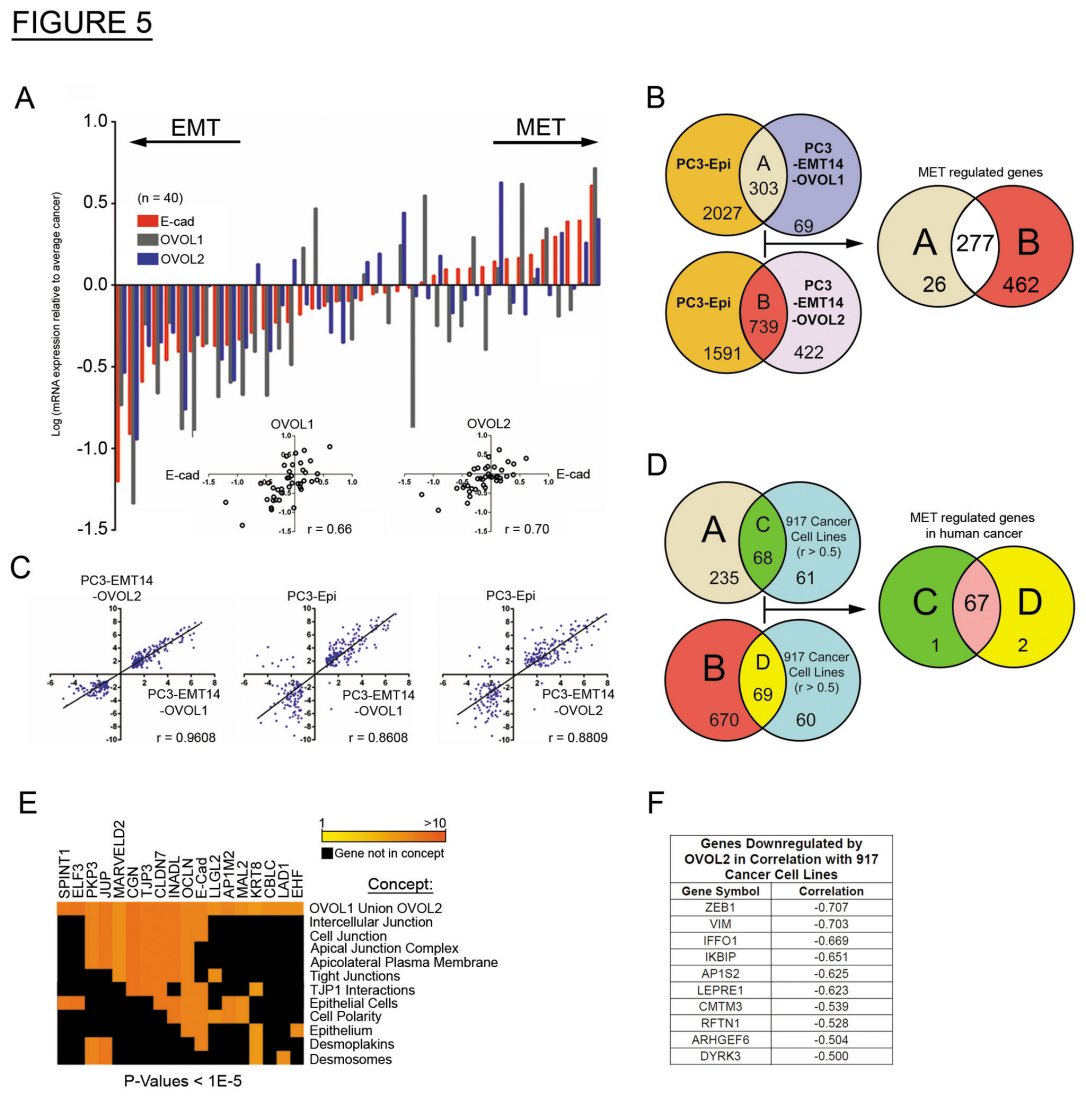
OVOL expression correlates with the epithelial cell state in multiple human cancer cell lines. (A) qPCR: cDNAs from human primary prostate cancer tissues (n = 40; Origene) was analyzed for the expression of E-cad, OVOL1 and OVOL2. The results were normalized to β-actin and shown relative to the average of all cancer samples for each gene as: . log ((Value of Gene (X) for a Sample) / (Value of Gene (X) for Average Cancer)). The sample values are shown in the dot plots and the correlation of OVOL1 or OVOL2 expression with E-cad was calculated (r). The graph depicts a representative experiment out of two with similar results. (B) Venn diagram: Depicts the RNA-seq results of differentially expressed genes common in the OVOL expressing cells and PC3-Epi, relative to PC3-EMT14. The intersection of A and B represents a common epithelial transcriptional signature of 277 genes. The RNA-seq data was analyzed from at least two biological replicates for each cell line. (C) Dot plot: Expression correlation analyses of the 277 genes identified in the epithelial signature from panel (B). Correlation is depicted between PC3-Epi, PC3-EMT14-OVOL1 or PC3-EMT14-OVOL2. (D) Venn diagram: Compares the results from panel (B) with the genes that correlate with the expression of the OVOLs in 917 human cancer cell lines. From the 129 genes that correlated (r > 0.5) with the OVOLs expression in the 917 cancer cell lines, 67 genes are induced by the expression of both OVOL1 and OVOL2 in PC3-EMT14 (C intersection D). (E) Heat map: ConceptGen analysis of the 67 gene-signature from panel (D) revealed a list of 18 annotated genes with functions related to the epithelial state of the cells. (F) Table: 45 genes negatively correlated with OVOL2 expression (r < -0.5) across 917 cancer cell lines. Among these 45 genes, the 10 shown are also downregulated by OVOL2 expression in PC3-EMT14. Note the TF ZEB1 and the mesenchymal marker vimentin (VIM) are the top genes in this list. See also Figure S5 and Tables S2, S3, and S4.

We next looked for consistent results using tissue-cDNA arrays from sections of human prostate cancer tumors (n = 40), by evaluating expression of E-cad, OVOL1, and OVOL2. To demonstrate the correlation between E-cad and OVOL-expression we normalized each sample value to the average value for all tumor samples. We observed strong correlation between E-cad and OVOL1 (r = 0.66) and OVOL2 (r = 0.7) in all tested prostate tumor tissues (Figure 5A).

Using Oncomine (Compendia Bioscience, Ann Arbor, MI) we assessed the generality of our results across multiple cancer cell lines. The Adai Cell Line (206 cancer cell lines, 27 cancer-types), Wooster Cell Line (314 lines, 107 cancer-types), and Barretina Cell Line (917 lines, 178 cancer-types) [14], all demonstrated high correlation in expression between the OVOL TFs and both E-cad and ESRP1, as well as other genes in the MET signature shown in Figure 1F (Figure S5A and Table S2). In addition, the 917 cancer cell-lines in the Barretina study show high correlation between the expression of OVOL1 and OVOL2 (r = 0.76).

Following-up on the expression differences observed between epithelial and mesenchymal cells in the microarray study, we used RNA-seq to explore differential gene expression across PC3-Epi, PC3-EMT14, PC3-EMT14-OVOL1 and PC3-EMT14-OVOL2 cells. OVOL expression induced profound changes in the cell transcriptome as compared to the parental mesenchymal PC3-EMT14 cells. A common gene signature for this transition revealed 277 genes that correlated with MET (Figure 5B). We observed a strong correlation in gene expression across the epithelial cells (Figure 5C) and changes in the top 100 genes are shown in Figure S5B. ConceptGen analysis [21] revealed enrichment for concepts consistent with MET (Table S3).

To test the roles of OVOL-TFs in MET, as well as the potential function of OVOL2 as a transcriptional repressor of ZEB1, we correlated our RNA-seq results with gene expression in multiple cancer cell lines (Barretina study). We only considered genes that showed a positive (r > 0.5) or negative (r < -0.5) correlation with OVOL-TF expression. For the positive correlation, we identified 129 genes that correlate with both OVOL TFs, suggesting a concurrent role in MET. Importantly, 67 of the 129 genes are induced by both OVOL1 and OVOL2, and show high correlation in E-cad expression (Figure 5D). ConceptGen analysis of this 67 gene-signature revealed a list of 18 annotated genes with functions related to MET (Figure 5E). These genes are involved in regulation of intercellular junctions, tight junctions, cell polarity, desmoplakins and desmosomes and genes related to epithelial-specific expression. Six of the 67 genes are not annotated in ConceptGen, including ESRP1 and EPCAM, both of which show high correlation with expression of both OVOLs. The negative correlation set includes 22 genes (Table S4). In this list we identified factors that induce EMT or are known markers of mesenchymal cells, including ZEB1 (r = -0.7), ZEB2 (r = -0.62) and Vimentin (VIM) (r = -0.7). Interestingly TGFβ1 (r = -0.53) negatively correlated only with the expression of OVOL2. Furthermore, from the genes that inversely correlate with OVOL2, 10 genes are downregulated by OVOL2 expression in PC3-EMT14 (Figure 5F). Notably, ZEB1 is the most negatively regulated gene in this list.

Alternative splicing is among the most important gene regulatory mechanisms in EMT-MET [22]. This post-transcriptional regulation acts in concert with other regulations to control transitions between the epithelial and mesenchymal states. Accumulating evidence suggests that alternative splicing is largely orchestrated by ESRP1 and ESRP2 [10,17]. Our study shows that the ESRPs are regulated by the OVOLs in mesenchymal cancer cells, and that their expression correlates with the OVOLs in the Barretina study: r = 0.76 with OVOL1 and r > 0.81 with OVOL2.

Interestingly, when analyzing the RNA-Seq results for genes that did not show overall expression changes, but showed significant isoform switching, we found that OVOL1 and OVOL2 induced post-transcriptional splicing of 15 and 237 genes, respectively. However, isoform switching of only five genes was regulated by both TFs: CD44, ENAH, IP6K2, LGALS8 and GPR126. Further comparisons between PC3-Epi and PC3-EMT14 cells identified 50 genes whose splicing regulation was induced by OVOL expression (Table 1). Three of five isoforms regulated by both OVOL TFs were also common to PC3-Epi cells: CD44, ENAH and IP6K2. Both CD44 and ENAH are regulated by ESRPs, showing differential splicing in epithelial versus mesenchymal cells [22]. Among the 50 splicing isoforms induced by OVOL TFs and also regulated in epithelial cells, 11 were expressed exclusively in either mesenchymal or epithelial cells (Table 1). Intriguingly, one of the genes showing a switch in isoforms, EPB41L5, is required for EMT during mouse gastrulation, and regulates E-cad post-transcriptionally (Figure S5C) [23,24]. This regulation of EPB41L5 suggests that splicing may play additional roles in OVOL-induced MET.

**Table 1:** Isoforms Regulated by the Expression of OVOL1 and OVOL2.

**NCBI Reference ID**	**Gene Symbol**	**PC3-EMT14**	**PC3-EMT14-OVOL1/PC3-EMT14-OVOL2**	**Fold Change (MET/EMT)**	**Isoforms up in MET**	**Isoforms up in EMT**
NM_001145356	CBWD1 §	0.60	6.24	10.41	+	
NM_001005911	IP6K2 §	25.84	7.55	0.29		+
NM_001001390	CD44 §§	2.11	12.91/12.86	6.07/6.11	+	
NM_001008493	ENAH §§	2.08	6.13/4.81	2.91/2.31	+	
NM_016351	ADAM22	1.00	0.40	0.4		+
NM_014324	AMACR	5.29	2.35	0.45		+
NR_036556	ANKRD54	0.99	0	0		+
NM_001010986	ATP11C	4.32	1.50	0.35		+
NM_174957	ATP2A3	0.26	0	0		+
NR_024597	C11orf73	0	0.46	INF	+	
NM_001001389	CD44	0.39	5.13	13.20	+	
NM_001185177	CES3	0	0.31	INF	+	
NM_203356	CTAGE5	0.55	0.13	0.24		+
NM_001085460	CTNND1	12.89	5.42	0.42		+
NM_133375	DIS3L	1.50	0	0		+
NM_004432	ELAVL2	1.45	0.37	0.26		+
NM_001135023	ELMOD3*	0.35	2.49	7.06	+	
NM_001135022	ELMOD3*	3.76	1.85	0.49		+
NM_203343	EPB41	0	0.97	INF	+	
NM_001184939	EPB41L5*	1.49	3.66	2.47	+	
NM_020909	EPB41L5*	1.74	0.62	0.36		+
NM_017848	FAM120C	1.86	0.92	0.5		+
NM_001015045	FAM13A	3.33	1.56	0.47		+
NM_001134456	FAM55C	5.55	2.65	0.48		+
NM_000802	FOLR1	0.06	0.32	5.44	+	
NM_001018100	GCOM1	1.14	4.52	3.96	+	
NM_001193322	IKBKE	0.90	0	0		+
NM_016291	IP6K2	6.10	0	0		+
NM_177535_1	MAGED4B	9.11	3.07	0.34		+
NM_145687	MAP4K4	22.74	10.81	0.48		+
NM_001042453	MST4	0	0.31	INF	+	
NM_022764	MTHFSD	1.67	0.75	0.45		+
NM_203318	MYO18A	2.38	5.78	2.43	+	
NM_001098623	OBSCN	0.22	0.67	3	+	
NM_001128629	PAK6	3.01	6.25	2.07	+	
NM_001166109	PALLD	1.84	6.80	3.69	+	
NM_001146105	PARP9	5.53	11.41	2.06	+	
NM_001184917	PCYT2	1.10	3.14	2.85	+	
NM_153321	PMP22	13.01	5.04	0.39		+
NM_182948	PRKACB	3.74	0.90	0.24		+
NM_001164758	PRKAR1B*	1.40	4.29	3.06	+	
NM_001164759	PRKAR1B*	5.13	2.19	0.42		+
NM_002840	PTPRF	1.69	6.52	3.85	+	
NM_144489	RGS3	5.16	1.43	0.28		+
NM_138484	SGOL1	0	0.41	INF	+	
NM_001135054	SIGIRR	15.50	6.13	0.4		+
NM_020428	SLC44A2*	5.89	22.83	3.88	+	
NM_001145056	SLC44A2*	28.71	5.32	0.19		+
NM_001104590	SLFN11	1.80	0.60	0.33		+
NM_001184749	SLITRK4	0.28	0.09	0.32		+
NM_001128210	SPRED2	4.30	0.37	0.09		+
NM_001198531	TCF7L2	2.05	0.74	0.36		+
NM_001167912	VEPH1	4.41	0	0		+
NM_014667	VGLL4	2.55	6.17	2.41	+	
NM_139119	YY1AP1	2.08	4.91	2.36	+	
NM_001145448	ZNF200	0.50	0.15	0.3		+

Isoform expression changes in PC3-EMT14-OVOL1 or PC3-EMT14-OVOL2 relative to PC3-EMT14 (p > 0.008). All the isoforms shown were also significantly changed in PC3-Epi relative to PC3-EMT14. Changes in isoform expression that are exclusive to PC3-EMT14-OVOL1 are marked with a single § Those that changed in both PC3-EMT14-OVOL1 and PC3-EMT14-OVOL2 are marked in with §§ (values presented as OVOL1-value/OVOL2-value). Isoforms singularly expressed in PC3-EMT14-OVOL2 are shown without any symbols, except those expressed exclusively in either EMT (mesenchymal) or MET (epithelial), represented by *

### OVOL-TFs induce MET and reduce invasive potential in a breast cancer model

**Figure 6:**
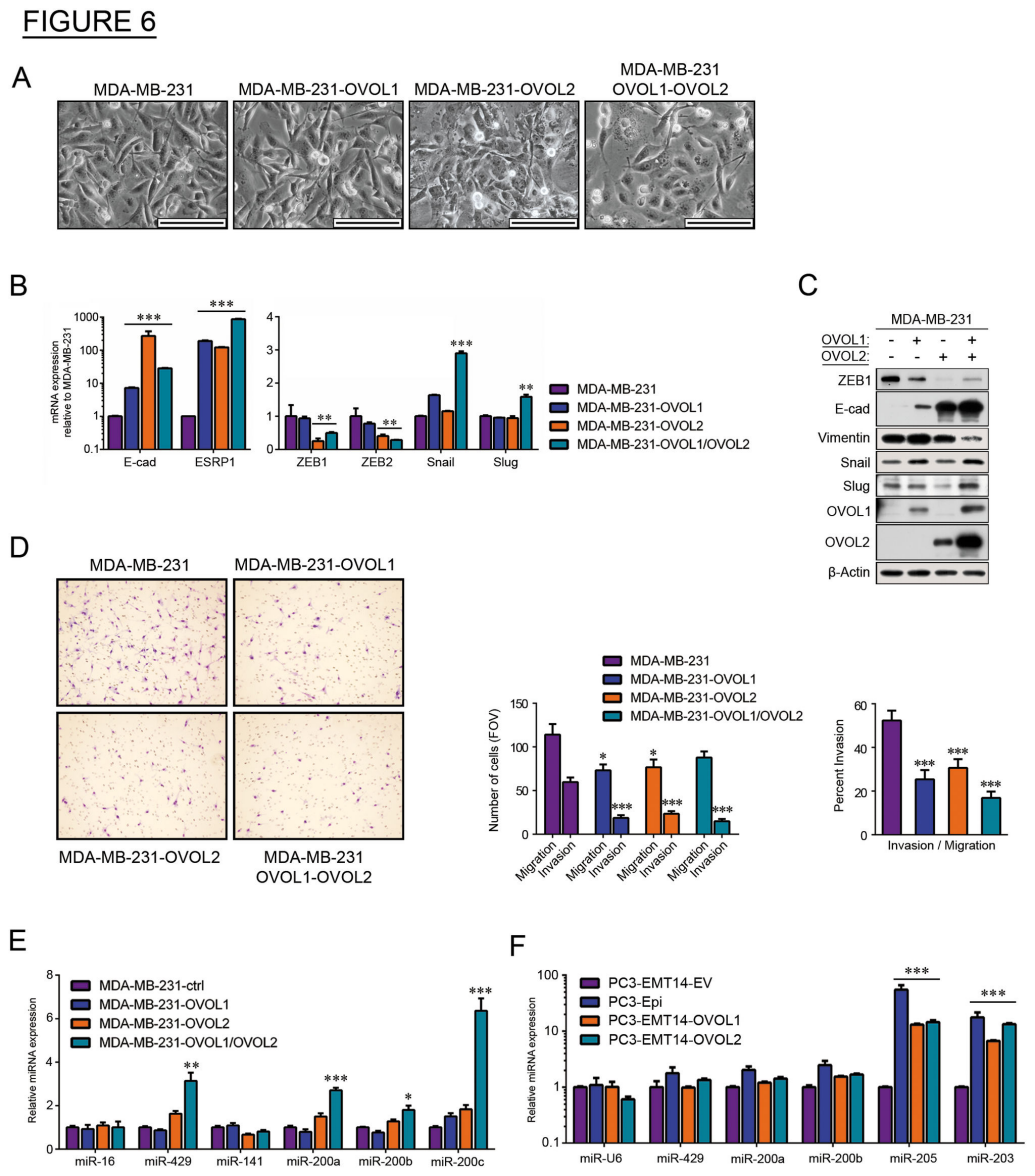
OVOL1 and OVOL2 induce MET in MDA-MB-231 breast cancer cells. (A) Bright-phase microscopy: morphology changes in the OVO-overexpressing MDA-MB-231 cells towards an epithelial phenotype. Breast cancer cells were transfected with OVOL1, OVOL2 or both TFs. Scales are 100 µm. (B) qPCR: Expression of the epithelial cell markers E-cad and ESRP1, and the EMT-inducing TFs ZEB1, ZEB2, Slug, Snail, and Twist1 in OVOL-overexpressing cells relative to control. Results were normalized to β-actin. (C) Immunoblot: Expression of EMT markers in MDA-MB-231 cells overexpressing OVOL1 and/or OVOL2 (represented by +/- in the table above the blot). (D) Invasion/migration assay: Representative images and graphs of cancer cell invasion using a Boyden chamber assay. Bar graphs depict the lower migratory and invasive potential of cells overexpressing the OVOL TFs. Percent invasion represents the ratio invading/migrating cells. The graph depicts a representative experiment out of three with similar results. (E) qPCR: miRNA expression in MDA-MB-231 cells overexpressing OVOL1, OVOL2, or both, relative to the control. Results were normalized to miR-U6. (F) qPCR: miRNA expression in PC3-Epi, PC3-EMT14-OVOL1 or PC3-EMT14-OVOL2 relative to the control (PC3-EMT14-EV). Results were normalized to miR-16. Graphs show mean +/- sem; p-values were calculated and represented as * p < 0.05; ** p < 0.01; *** p < 0.001. The qPCRs and immunoblots are representative of two independent experiments with similar results.

To test these results in a context outside of prostate cancer, we next used the poorly differentiated (mesenchymal-type), highly invasive, breast carcinoma MDA-MB-231 cell line [25]. These cells were transduced with lentiviruses expressing OVOL1, OVOL2, or both. Cells expressing OVOL2 or both showed significant changes in morphology, shown by a decrease in mesenchymal (elongated) cells and an increase in epithelial-like cells (Figure 6A). To test whether the observed morphological changes were related to MET, we used qPCR to measure the expression of E-cad, ESRP1, and the 5 TFs previously tested: ZEB1, ZEB2, Snail, Slug and Twist [2]. Consistent with MET, we observed significant increases in E-cad and ESRP1 expression in all OVOL-expressing cells (Figure 6B, left panel), while ZEB1 and ZEB2 expression significantly decreased (Figure 6B, right panel). Changes in Snail and Slug did not correlate with MET, and Twist mRNA was undetected. Consistent with the expression changes, Western blot revealed a drop in ZEB1 and an increase in E-cad proteins in OVOL-expressing cells, along with an accentuated increase with OVOL2 (Figure 6C). Transduction with both OVOLs increased the levels of each of the OVOL proteins, compared to single-transduced cells, suggesting a cross-regulation of these proteins in these cells.

As in the prostate cancer model, we used Boyden chamber assay to assess invasion in MDA-MB-231 cells expressing OVOL1, OVOL2, or both. In the OVOL-expressing cells, percent invasion declined and the most striking decrease was observed in cells expressing both TFs, indicating OVOL-mediated MET attenuates the invasive phenotype (Figure 6D).

Given evidence of reciprocal feedback loops between ZEB1/2 and miR-200 family members in EMT-MET transformations [7,26], we explored the contribution of miR-200s as potential inducers of epithelial differentiation in the breast and prostate cancer models. In MDA-MB-231 cells expressing the OVOLs, we used qPCR to assess expression of miR-200 family members (miR-200a, miR-200b, miR-200c, miR-141, miR-429, miR205, miR-203), relative to controls miR-U6 and miR16. The double-transduced cells showed greater than 2-fold increases in miR-200a, miR-200c, and miR-429 (Figure 6E), while only modest changes were seen in the single-transduced cells. In particular, OVOL2-expressing cells showed small changes in miR-200s (less than 2-fold in all cases), which did not correlate with changes observed in E-cad and ZEB1 protein (Figure 6C). In our prostate cancer model, we tested the expression of miR-200s in PC3-EMT14 and the epithelial cells: PC3-Epi, PC3-EMT14-OVOL1 and PC3-EMT14-OVOL2. We observed significant upregulation of miR-203 and miR-205 in epithelial cells (Figure 6F). Comparing these observations to the MDA-MB-231 model, we found no general correlation between the expression of OVOL and the members of miR-200-family across cell types, suggesting that regulation of miRNA-200s is cell-type specific. In addition, these findings suggest that the OVOLs may indirectly regulate some miR-200s, which could also contribute to the stability of the epithelial state.

## Discussion

The data presented here reveals a novel role for OVOLs as important regulators of MET in cancer cells. Using a combination of in vitro and in vivo experiments, as well as data from cancer databases, we show that OVOLs orchestrate a transcriptional and splicing-regulatory program that mediates MET, resulting in decreased cancer cell invasiveness. Furthermore, we describe a critical role for the OVOLs as regulators of cancer cell metastasis, by repressing ZEB1 and inducing ESRP1.

Tumor cells’ ability to invade surrounding tissues and disseminate to distant organs requires them to activate a program allowing them to undergo EMT, detach from the primary tumor, enter circulation, and transit to a secondary site. EMT is thought to be a transient program, where changes in extrinsic signaling factors induce mesenchymal cells to revert back to the epithelial state [4]. MET has been associated with cancer cells’ ability to adapt to a new site, proliferate, and form macrometastases. This hypothesis is supported by the observation that metastases originating from epithelial cancers show a high degree of differentiation towards the epithelial phenotype. Meanwhile in cancer patients, heterogeneous differentiation has been observed even in metastases from a single patient [27]. In our studies of prostate cancer metastasis, we discovered that some cells that form metastases do not undergo MET, and that mesenchymal cells can proliferate to develop mesenchymal-type solid tumors (Figure 3C and Figure 4E).

Usually, activation of EMT involves signaling between tumor cells and neighboring stromal cells [28]. To determine the mechanisms of MET; we developed a novel in vitro model of prostate cancer that mimics interactions within the prostate tumor. First, we generated a prostate cancer epithelial cell line (PC3-Epi) that stable maintained the epithelial phenotype in culture, while preserving their potential plasticity (EMT/MET). Consequently, these cells predominantly originated epithelial tumors, though they also demonstrated their plasticity to develop into mesenchymal tumors in vivo via EMT (Figure 3C). We showed that interactions between epithelial prostate cancer cells with M2-type macrophages resulted in EMT, as well as the upregulation of the EMT-inducing TF ZEB1. Subsequently, we used this model to interrogate the mechanisms that maintain the mesenchymal or epithelial phenotype. Microarray analysis of three different EMT cell subpopulations (Figure 1A-C) showed that other EMT-inducing TFs like Twist, Snail or Slug were not commonly upregulated. Furthermore, it is known that TGFβ on its own rarely induces EMT, despite being one of the major factors that induces the EMT cell transformation [29]. For example Scheel et al. used an EMT induction cocktail in combination with TGFβ1 to induce EMT in epithelial HMLE cells [30]. Importantly, consistent with our findings, the resultant cells demonstrated a significant induction of the ZEB1/ZEB2 TFs.

It has been suggested that ZEB1 mediates EMT-MET plasticity through a feedback loop including the microRNA-200 family [26]. However, the regulation of MET by MET-inducing TFs is under-studied. In our model, we found that ZEB1 silencing resulted in MET induction and the upregulation of four TFs in mesenchymal cancer cells (Figure 1F). Therefore, we investigated their potential roles in MET. We identified that the expression of either OVOL1 or OVOL2 represses ZEB1, and also discovered that ZEB1 regulates their expression (Figure 2A-E). Both OVOLs, in turn, induce ESRP1, whose function has been demonstrated to play a role in MET [9,10].

OVOLs are zinc finger TFs that control gene expression in various developmental processes, in multiple organisms. These factors have been shown to reside downstream of key developmental signaling pathways, such as Wnt and BMP/TGFβ [11,12]. These same pathways collaborate in the activation of EMT [30]. However little is known about the role of the OVOLs in cancer or their regulation of MET. We demonstrated that in primary tumors of prostate cancer patients the expression of OVOL1 and OVOL2 highly correlate with E-cad expression, a critical marker of the differentiation state of these tumors (Figure 5A). We analyzed the gene expression changes induced by the OVOL-TFs (identified by RNA-Seq) relative to the control PC3-EMT14 cells. From this analysis, we identified the genes that changed over 2-fold. We compared this list with the 50 genes signature, which comprises the changes induced by ZEB1-shRNA in the EMT cells (Figure 1F). The results demonstrated that 43 out of 50 genes also changed upon OVOL1 expression, which closely correlates with the MET cellular response induced by ZEB1-shRNA. Likewise, OVOL2 induced the expression changes in 45 out of these 50 genes. These findings suggest that the majority of the changes induced by ZEB1-shRNA in relation to MET are also induced by the expression of the OVOL-TFs, further supporting the significance of the cross regulation of ZEB1 and the OVOL-TFs in MET.

To expand the investigation of the role of the OVOLs in regulation of MET in human epithelial cancers, we analyzed several studies compiled in the Oncomine database. Our analysis revealed that expression of both OVOLs highly correlates with expression of E-cad, ESRPs, as well as factors associated with the epithelial phenotype that were upregulated in our prostate cancer model (Figure S5B). Furthermore, this analysis identified the same set of genes to correlate positively with either OVOL1 or OVOL2 expression (r > 0.5) in 917 cancer cell lines. From the list of factors showing a correlation higher than 0.74 (Table S2), we found that 18 out of 24 genes were induced by both OVOLs in mesenchymal prostate cancer cells. Additionally, a similar database analysis demonstrated that both OVOLs negatively correlate with the expression of ZEB1 and Vimentin (Table S4). The negative correlation between expression of the OVOLs and ZEB1 in multiple cancers indicates that the function of the OVOLs may not be restricted to prostate cancer. We validated this mechanism in another cancer-type by expressing the OVOL-TFs in the poorly differentiated breast cancer MDA-MB-231 cells. Similar to our finding in prostate cancer cells, expression of OVOLs induced MET and high ESRP expression, while significantly decreasing ZEB1 expression (Figure 6A-C). In this model the maximum degree of MET was achieved by expression of both OVOLs. Similar to the prostate model, OVOL-expression attenuated the invasive potential of the MDA-MB-231 cells. These results are consistent with previous findings showing that overexpression of ESRPs attenuates EMT and malignancy in MDA-MB-231 cells [9]. Horiguchi et al. further demonstrated that the expression of ESRPs in human breast cancer tissues inversely correlated with progression and that high ZEB and low ESRP expression characterize the more aggressive (‘basal like’) subtype of breast cancer. Furthermore, consistent with our data, Snail, Slug, and Twist did not show significant inverse correlation with ESRPs. In that study, expression of ESRPs partially induced MET in the MDA-MB-231 cells, mainly through upregulation of E-cad. Using the same cell line, we show that increased E-cad is achieved through overexpression of both OVOL-TFs, which also results in downregulation of the mesenchymal marker Vimentin. Our finding of OVOL regulation of ESRP and ZEB1 further supports the significance of their function in MET.

The transcriptional repressor function of OVOL2 on ZEB1 is supported by the mRNA upregulation of ZEB1 upon OVOL2 depletion (Figure 2G), by the direct interaction of OVOL2 with ZEB1 promoter region (ChIP assay, Figure 2I-J), and by the depletion of ZEB1 protein and mRNA upon overexpression of OVOL2 (Figure 2C-E and Figure 6B-C). The finding that OVOL2 depletion (but not OVOL1) induces the upregulation of ZEB1-mRNA (Figure 2G), suggests that OVOL2 plays a predominant role in the regulation of ZEB1. However, the direct regulation of ZEB1 by OVOL1 is not demonstrated and OVOL1 might regulate ZEB1 indirectly. Further investigation would be necessary to address this issue. Nevertheless, the experiments in MDA-MB-231 cells demonstrated that the independent expression of each OVOL induced MET and led to depletion of ZEB1 mRNA and protein, while combined expression of both OVOLs further induced MET (Figure 6C). On the other hand, depletion of both OVOL1 and OVOL2 is necessary to induce partial EMT in PC3-Epi cells (Figure 2H). Furthermore, OVOL1 and OVOL2 are co-expressed in multiple cancer cell lines and they induce similar gene expression profiles consistent with MET (Figure 5B-E), which indicate analogous functions. Altogether, these findings suggest that they could complement each other in achieving maximum induction of MET.

Alternative mRNA splicing allows cells to create protein isoforms with different functions from a single gene. Cancer cells take advantage of this mechanism to produce proteins that contribute to tumor progression, invasion, and metastasis [31]. Thus, alternative-splicing mediated by ESRPs is a hallmark of epithelial cell differentiation, and activation of EMT produces isoforms associated with metastasis [22]. Warzecha et al. showed that loss of ESRP1 induced some of the phenotypic and protein expression changes related to EMT, suggesting that these changes are due to alternative splicing-mediated isoform switching [32]. As MET is essentially the opposite of EMT, the activation of this program requires the expression of ESRP1. Expression analysis by RNA-Seq in our model demonstrated that OVOLs activate a splicing program that induces isoform variants specific to the epithelial state (Table 1). Several genes showed significant isoform switches between MET and EMT, even though overall gene expression did not change (Table 1). Many of these isoforms (including CD44, ENAH, p120-Catenin, EPB41L5) show direct correlation with both MET-EMT and metastasis, in multiple cancer types [10,22]. Other genes demonstrated changes both in gene and isoform-specific expression. Altogether these findings suggest that the post-transcriptional control of gene expression is a complement to the transcriptional regulation programs that drive the MET/EMT transformations. Current approaches to discover meaningful changes in alternative splicing in different models involve manipulations with ESRPs or EMT-inducing TFs [33]. Elucidation of OVOL-mediated splicing regulation represents a novel approach to identify MET and EMT-biomarkers that may help clinicians to define tumor aggressiveness.

It has been suggested that MET regulation involves miR-200s, whose expression directly affects cell proliferation [4]. In our experiments with MDA-MB-231 cells, MET did not always correlate with expression of miR-200s (Figure 6C and 6E). However, the expression of both OVOL TFs resulted in the induction of miR-429, miR-208 and miR-200c. This suggests that, in addition to the previously described mechanism, the OVOL-mediated MET may involve regulation of the miR-200 family. This additional regulation could explain why more than 3-fold upregulation of ZEB1 mRNA is required to induce EMT in the epithelial prostate cancer PC3-Epi cells (Figure 2G and Figure S2A, S2C-D). The EMT induction requires a shift in the balance between the MET and the EMT-inducing factors and this could only be achieved by a significant increase in the ZEB1 expression. The role of miRNAs in this mechanism requires further investigation.

**Figure 7:**
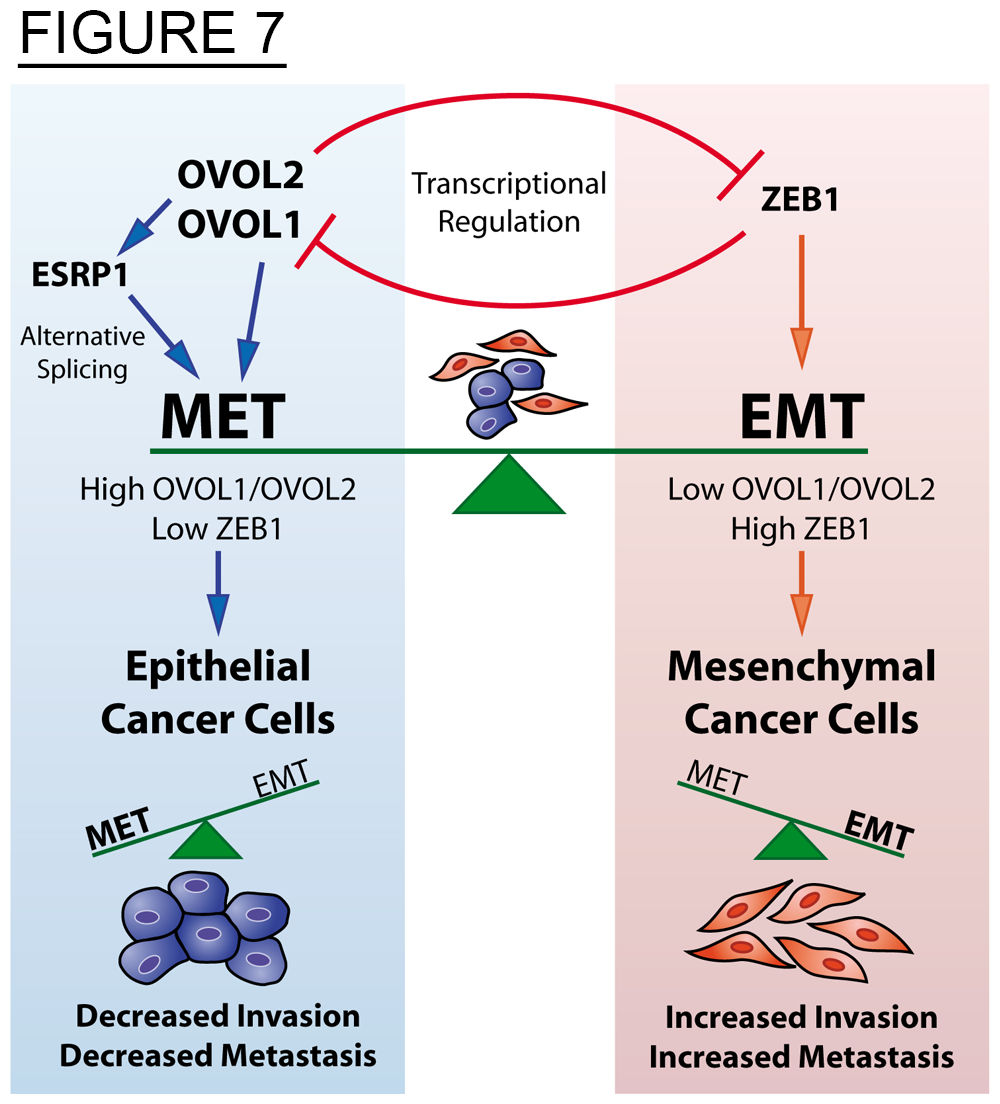
Proposed model of EMT/MET balance in human cancer. In human cancer cells the mesenchymal and epithelial states are induced and maintained by transcriptional and post-transcriptional (splicing) regulatory programs. These programs are controlled by the feedback regulation between the OVOL and the ZEB1 TFs, critical inducers of MET and EMT respectively. In addition these TFs control the expression of ESRP1, a key-splicing regulator activated in MET and repressed in EMT. Therefore high OVOL and low ZEB1 stabilize the epithelial state decreasing cancer cell invasion and metastasis, and vice versa for the mesenchymal state.

We propose that the mesenchymal or epithelial state of cells is controlled, in part, by a regulatory feedback loop between the OVOLs and ZEB1, as well as a mechanism induced by the OVOLs to regulate mRNA splicing through ESRPs (Figure 7). We hypothesize that when environmental conditions favor expression of OVOLs, cells will transition towards the epithelial state, where they are stabilized by regulatory feedback loops. These epithelial cancer cells will have a reduced invasive and metastatic capacity and show resistance to EMT. Alternatively, low expression of OVOLs and high expression of ZEB1 will induce EMT. When this mechanism is activated, the mesenchymal state could be stabilized by regulatory feedback, making the cells non-dormant and capable of progressing through a colonization step. The result would be a highly metastatic tumor in a predominantly mesenchymal state. Our findings in xenograft models of prostate cancer further validate our hypothesis by showing that the cancer cells demonstrate some tumor plasticity, while the mesenchymal or epithelial phenotypes are predominantly maintained as their states are stabilized by the feedback loops between the OVOL-TFs and ZEB1. This regulation may characterize the great majority of cancer cells, as demonstrated by the high correlation between the OVOL-TFs, ESRP1 and E-cad in addition to their inverse correlation with ZEB1 and vimentin in 917 cancer cell lines (Figure 5F and Figure S5A). In cancer patients, tumors may occur as a mix of differentiated and undifferentiated cells (Figure 3D). Together, our data improve our understanding of cancer metastasis by revealing crucial TFs that induce MET in cancer cells and regulate the balance between EMT and MET. Further investigation of the mechanisms that regulate EMT-MET might lead to novel therapies that halt metastasis.

In summary, the findings presented here suggest a new mechanistic model of metastatic progression in which epithelial-mesenchymal cancer cell plasticity is mediated by the crucial function of the OVOLs. An assessment of OVOL to ZEB1 expression ratio may identify the trigger point for EMT-MET in various tumor cells. However, this ratio might change, depending on the specific cancer cell’s susceptibility (genetic/epigenetic profile) to undergo EMT. Our future studies will address whether OVOLs regulate additional EMT-inducing TFs, and investigate if alternative feedback loops are activated. It is tempting to speculate that the mesenchymal state will not be stabilized if it is not mediated by TFs that activate a reciprocal feedback loop.

## Materials and Methods

### Ethics Statement

All animal work was carried out in strict accordance with recommendations in the Guide for the Care and Use of Laboratory Animals of the National Institutes of Health. University of Michigan Committee on Use and Care of Animals approved the protocol (UCUCA- Approval Number-08434). All surgery was performed under ketamine/xylazine or isoflurane anesthesia and all efforts were made to minimize animal suffering.

### Cell lines

Prostate cancer PC3 and DU145 and breast cancer MDA-MD-231 cell lines were obtained from ATCC. These cells and all derived cell lines were cultured in RPMI-1640 (Invitrogen), and supplemented with 10% fetal bovine serum (FBS) and 1% Antibiotic/antimycotic (Invitrogen). Cells were passaged at 80% confluency using 0.05% trypsin (Invitrogen).

### Generation of stable epithelial PC3-Epi cells

PC3 cells were transfected with pLentilox-EV-Luc luciferase expression vector (University of Michigan Vector Core). Luciferin was added to culture plates and bioluminescent imaging was done. Colonies were selected based on two criteria: bioluminescent intensity and epithelial cell morphology. Selection was repeated twice to ensure the majority of the cells had luciferase activity. A stable epithelial population was obtained and further characterized by Western blot analysis.

### Isolation of stable EMT cells from macrophage co-culture

Human peripheral blood mononuclear cells (PBMC) were isolated as, previously described, from healthy donors, according to HUM# 0024137 (University of Michigan Health System) [34]. CD14^+^ monocytes were isolated using CD14-magnetic beads (Miltenyi; Cat no 120-000-305) as per manufacturer’s instructions. The CD14^+^ cells were forced to differentiate into M1 or M2 macrophages upon stimulation with INFγ (R&D; 285-IF/CF) or interleukin (IL)-4 (Humanzyme; HZ-1004) respectively (100 ng/ml) for 36-42 hours. Previous studies have shown this to be sufficient time to induce macrophage differentiation into M1 or M2 types [35]. Following the initial 36-42 hour incubation, the media was changed and INFγ concentration was reduced to 2 ng/mL. The resulting M1 or M2 cells were co-cultured with 3x10^5^ highly epithelial prostate cancer cells (PC3-Epi) for 4 days. Cells were passaged 3 times through trypsinization and 10^4^ cells were plated onto 10 cm tissue culture plates. A parallel culture of M2 macrophages was included to assure that no macrophage will survive after 3 trypsinization passages. Populations showing change in morphology were further isolated (PC3-EMT1, PC3-EMT12, and PC3-EMT14) and characterized by Western blot and gene expression analyses.

### Lentiviral constructs and transduction

#### Expression constructs

Lentiviral plasmids were created with the ViraPower™ II Lentiviral N-Lumio™ Gateway® Expression System (Invitrogen) cloning system, using manufacturer specifications. Premade entry clones were purchased from Thermo Scientific/Open Biosystems for *OVOL2* (clone ID: 5098), *IRF6* (clone ID: 2399) and *TSPAN8* (clone ID: 100073674). Destination clone pLenti6.2/C-Lumio™/V5-DEST (Invitrogen; Cat no: K370-20) was used to construct the lentivirus plasmids and University of Michigan Vector Core packaged plasmids into lentivirus. Overexpression constructs for *OVOL1* (clone ID: PLOHS_100067324), *EHF* (clone ID: PLOHS_100070253), and *ANKRD22* (clone ID: 100072173) were purchased from Thermo Scientific/Open Biosystems.

#### shRNA constructs

*ZEB1-shRNAs*, designated sh2, and sh4 (clones IDs: V3LHS_356184, and V2LHS_116659, respectively), *OVOL1*, designated a, b, and c (clones IDs: V3LHS_ 400419, V3LHS_305031, V3LHS_ 47894, respectively) and *OVOL2*, designated e, f, g, (clones IDs: V3LHS_354271, V3LHS_354272, and V3LHS_60547, respectively), non-silencing control (Cat no: RHS4348) (OpenBiosystems), and scramble control (Lenti-pGipZ-scramble-VSVG; University of Michigan Vector Core).

#### Transduction

Cells were transduced with lentiviruses and selected using manufacturer recommended concentrations and protocols.

### RNA isolation

RNAs were isolated from cells at ~80% confluency using an RNeasy kit (Qiagen) and treated with DNase to remove genomic DNA (Qiagen). RNA quality and concentration was determined by accudrop and nanodrop (University of Michigan Microarray core).

### miRNA isolation

miRNA was isolated from cells at ~80% confluency using an miRNeasy kit (Qiagen Cat no: 217004) following manufacture’s protocol. RNA quality and concentration was determined by accudrop and nanodrop (University of Michigan Microarray core).

### Microarray

Extracted mRNA was subjected to microarray analysis using standard protocols by the University of Michigan Microarray core for the GeneChip Human U133 Plus 2.0 (Affymetrix). Gene expression analysis was conducted by University of Michigan Microarray core using Bioconductor’s “Limma” package. The microarray data “Expression profile from PC3-Epi and derived cell lines” is accessible with the GEO ID: GSE43489.

### qPCR

cDNA was prepared using high capacity cDNA reverse transcription kit (Invitrogen). Quantitative analysis was performed using TaqMan Gene Expression Assays (Applied Biosystems) with ABI 7900 HT. The following primers/probes were used: *β-actin* 4352935E, *OVOL1* Hs00190060_m1, *OVOL2* Hs00221902_m1, *ZEB1* Hs00232783_m1, *E-cad* (*CDH1*) Hs01023894_m1, *TWIST1* Hs01675818_s1, *ZEB2* Hs00207691_m1, *Snail* (*SNAI1*) Hs00195591_m1, *Slug* (*SNAI2*) Hs00950344_m1, and *ESRP1* H`s00214472_m1.

Relative Expression Calculations: In the qPCR, the target of interest in each sample is measured using three biological replicates. The Ct value for each biological replicate is calculated as an average of three technical replicates. Then the Ct value of each biological replicate is normalized to β-actin by subtracting it from the corresponding Ct value of β-actin (-ΔCt). The two groups of interest are compared using a Student’s t-test. The values plotted in the graph are the average of the base 2 anti-log transformations of -ΔCt for the biological replicates of interest divided by the average of the base 2 anti-log of -ΔCt for the reference group. The standard errors of the mean are determined from these 3 values of the biological replicates each divided by the mean for the reference group (control cell).

TaqMan MicroRNA Assays: *miR-200c* TM: 002300*, miR-200a* TM: 00502*, miR-429* TM: 001024*, miR-205* TM: 000509*, miR-141* TM: 000463*, miR-16* TM:000391*, miR-200b* TM: 002251*, miR-U6* TM: 001973, *miR-423-5p* TM: 002340*, miR-34b* TM: 000427*, miR-34a* TM: 000426, and *miR-34c* TM: 000428. Normalization was done to either miR-U6 (MDA-MB-231) or miR-16 (PC3-EMT14) and graphed relative to controls specified for each case.

Origene TissueScan™ Prostate Cancer cDNA Array II (HPRT302) was used as per manufacturer recommendations. Samples were normalized to β-actin, and the average for each gene was calculated among all cancer samples (average cancer, n = 40). Each sample is plotted as a log_10_ of the normalized value relative to the average cancer. Correlation graphs of OVOL1 and OVOL2 relative to E-cad values were constructed and Pearson coefficients were calculated using Prism GraphPad.

### Western Blot and Flow Cytometry

Protein extracts were prepared using cell lysis buffer (Cell signaling; Cat no 9803S) with protease inhibitor (Thermo Scientific; Cat no: 78410) and samples were analyzed by Western blot using Invitrogen specified instructions. Western blots were performed using anti-E-cad (cat no: 3195), anti-ZEB1 (Cat no: 3396), anti-Vimentin (Cat no: 5741), anti-Slug (Cat no: 9585), anti-Snail (Cat no: 3879) (Cell Signaling), and anti-OVOL1 (Cat no: 14082-1-AP) (Proteintech Group). Anti-β-actin (Cell Signaling; Cat no: 4970) was used as loading control.

Flow cytometric analysis of PC3-Epi, PC3-EMT14 and PC3-Epi-TSPAN8 were conducted as previously described [34] using the FITC-anti-human CD326 (EpCAM) antibody (Clone 9C4, BioLegend; Cat no: 324203) or the APC-anti-human TSPAN8 (R&D; Cat no: FAB4734A).

#### Microscopy

Bright field: Images were obtained using phase contrast on the Olympus IX71 with a mounted Olympus DP71 camera.

### Invasion/Migration assay

Cells were trypsinized, washed once with 10% FBS, then twice with serum free media. 5*10^4^ cells/well were seeded in serum-free in triplicate on top of BD Matrigel™ Invasion Chambers (cat no: 354480) or uncoated control chambers. As per manufacturer specifications 0.5% FBS media was placed in the lower chamber. Staining was performed using Protocol HEMA 3 staining kit (Cat no 122-911). Cell invasion was assessed and quantified by averaging the counts from 5 fields of view per well for each replicate. Cell migration was evaluated by counting the cells that migrated to the bottom of the uncoated insert chamber (control).

### Mouse injections

ICI and subcutaneous injections with PC3-EMT12, PC3-EMT14, and PC3-Epi were performed in NSG mice (Jackson Laboratory) between 6-8 weeks of age. ICI studies using PC3-EMT14, PC3-EMT14-OVOL1 and PC3-EMT14-sh4 were done using CB17 SCID (Jackson Laboratory) male mice, between the 6-8 weeks old. Subcutaneous injections were performed using 2*10^5^ cells in 200 mL matrigel:DPBS (1:1 ratio). Mice then received injections in hind-flank, and were imaged weekly. Intra-cardiac injections were performed using 2.5*10^5^ cells in 0.1 mL of DPBS. Mice were anesthetized using isoflurane. Successful injections were determined based on initial bioluminescent imaging tumor locations. Mice with high bioluminescent signature in areas around or in the heart during the first 2 weeks were deemed unsuccessful, and removed from study. Orthotopic prostate injections were performed on male NSG mice (NOD.Cg-*Prkdcscid Il2rgtm1Wjl*/SzJ, Jackson labs) aged 10-13 weeks. 3*10^5^ luciferase-positive PC3-EMT14, PC3-EMT14-OVOL1 or PC3-EMT14-OVOL2 cells were injected into the dorsal prostate via laparoscopic surgery. The mice were imaged for bioluminescent signal weekly on an IVIS 200 and monitored for overall health and metastasis formation.

### In vivo bioluminescent analysis and histology

Bioluminescent imaging (University of Michigan Cellular molecular imagining core) was performed using the Xenogen IVIS 200 and histology preparation was done as previously described [19]. A pathologist from the University of Michigan Pathology Core for Animal Research analyzed the Immunohistochemistry (IHC) sections from mouse and human tissues. IHC antibodies: *anti-ZEB1* (Sigma, Cat no: HPA027524), and *anti-E-cadherin* (Abcam; Cat no: ab40772).

#### Chromatin Immunoprecipitation

We used Invitrogen’s MAGnify™ Chromatin Immunoprecipitation System (Cat no: 49-2024) following manufacturers protocol, except that antibodies and the chromatin DNA were incubated overnight. 5 µg of anti-OVOL2 (Santa Cruz Biotechnology, Cat no: sc-85803 X) or anti-V5 (abcam Cat no: ab9116) antibodies were used per sample. For the analysis of the chromatin DNA fragments by qPCR we designed custom primers/probes using Applied Biosystems’ Primer Express software (Table 2).

**Table 2:** Primers/probes designed using Applied Biosystems’ Primer Express software for the qPCR analysis of the immunoprecipitated chromatin DNA.

Names	Forward Primer	Reverse Primer	Probe
(-)4848	CTCAGGTAGAGGAACGAAGCTAGAG	GCTTTGGCAAATGATCAGAGAA	CAGGGAATAGAATAAATATC (MGB)
(-)3503	TTCCTGACTCAATGACTGCAGAA	CCCATAAAGCCGCTACTCAATT	CCATTGCCCTCCTTTGTTCCACGG (TAMRA)
(-)2012	CAGGCCAGAAAGAGAACATGAGA	TTTCCAGCTCTATCACACATTTTACC	AATGCTATTTGTAATACCTCC (MGB)
(-)1389	AAATCCTGCCATAGAAGTGACAAAA	GTGACCGGAGTATTGGAAATAACG	TATTGAGCACCTACTATGTGTCAT (MGB)
(-)765	GGTATTACTCATTCCGCTCTACTAAGG	CCGGGATGGGAAGTGACTT	TTTCAATCCAGCTGAAGTT (MGB)
(-)115	CAAGGTTCCGGCCGTAGA	CCCACCGCACCTGGTTTA	AAAGCCGGGAGTGTC (MGB)
(+)382	CGCCTCCCTGGACCGTTA	CGCCGAAGGGCACAAGA	AGCGGCTGTTGCTT (MGB)

### RNA-seq data analysis

Sequencing was performed by the UM DNA sequencing core, using the Illumina Hi-Seq platform to generate 50 base, paired-end reads. We downloaded and concatenated the individual reads files to correspond with individual samples. These fastq files are GEO dataset (GSE48230). We aligned the reads to the reference transcriptome (UCSC hg19) using TopHat2.0.2, which is part of the tuxedo next-generation sequencing data analysis suite [36,37]. We used default parameter settings with the exception that we specified “–b2-very-sensitive”. We used FastQC (http://www.bioinformatics.babraham.ac.uk/projects/fastqc/) to assess a range of quality measures, and found overall very good quality aligned reads in each sample. We then used CuffDiff2.0.2, also part of the tuxedo suite, to assess differential expression between groups (PC3-EMT14, PC3-EMT14-OVOL1, PC3-EMT14-OVOL2, and PC3-Epi), using the UCSC hg19.gtf transcriptome, with -u, -N, --compatible-hits-norm, and -b (relative to the UCSC hg19.fa) parameter settings. We used a locally derived Perl script to identify genes/transcripts as being differentially expressed if they showed: “OK” test status AND FDR ≤ 0.05, AND fold change (≥ 2.0 or ≤ 0.50).

### Enrichment Analysis

We submitted various gene sets to ConceptGen [21] for enrichment analysis. ConceptGen tests for enrichment across a broad set of annotations, using a modified Fisher Exact test to assess significance of enrichment.

### Statistics

We used a one-tailed Student’s t-test where applicable (qPCR with overexpression and shRNA, when the results were predicted before performing experiment). In cases where a one-tailed test was not appropriate, a two-tailed Student’s t-test was used (e.g. Mouse studies, miRNA and ChIP qPCR, invasion/migration assay). All error bars represent ±SEM. Statistical analysis was performed using Prism GraphPad. A value of P < 0.05 was considered significant.

### In vivo statistics

Tumor growth for each model was analyzed using a linear mixed model. The natural log transformed ROI was fit using a random slope and intercept. Each model was found to have a 1^st^-degree heterogeneous autoregressive covariance structure. Type 3 effects for the model and multiple-comparison adjusted least square means are given for the comparisons of interest for each experiment. Additionally, the time by cell line interaction was ‘sliced,’ in the sense that each comparison was also examined at each time point. For the survival component of each experiment, Kaplan-Meier plots were created and log rank values for these plots are reported. Pairwise log rank values adjusted for multiple comparisons for the comparisons of interest were also completed. A value of P < 0.05 was considered significant.

## Supporting Information

Figure S1 **Macrophages induce EMT in epithelial prostate cancer PC3-Epi cells.** Related to Figure 1.
(A) Schematic: Human CD14^+^ blood monocytes (3x10^6^ cells) were isolated from healthy donors and induced to differentiate into M1 or M2 macrophages upon stimulation with INFγ or interleukin (IL)-4 respectively (100 ng/ml) for 36-42 hours. Next, these cells were co-cultured with 3x10^5^ highly epithelial prostate cancer cells (PC3-Epi) for 4 days.
(B) Immunoblot: Shows downregulation of E-cad with the concomitant upregulation of vimentin in the cancer cell population from the co-culture with IL-4-treated CD14^+^ macrophages as compared to controls. The immunoblot is representative of two independent experiments with similar results.
(C) Flow Cytometry: Depicts a decrease in the cell surface expression of the Epithelial Cell Adhesion Molecule (EpCAM) in the stable mesenchymal PC3-EMT14 prostate cancer cells compared to the parental epithelial PC3-Epi cells. The results are representative of two experiments.
(TIF)

Figure S2 **OVOL and ZEB TFs are inversely regulated in the stable epithelial (PC3-Epi) and the stable mesenchymal (PC3-EMT14) prostate cancer cells.** OVOL2 binds to ZEB1 promoter. Related to Figure 2.
(A) qPCR: mRNA expression in PC3-EMT14 relative to PC3-Epi prostate cancer cells.
(B) Flow Cytometry: Depicts the cell surface expression of the transmembrane protein Tetraspanin-8 (TSPAN8) in the epithelial PC3-Epi cells transduced with a TSPAN8 expression lentivirus and compared to the parental PC3-Epi cells.
(C) qPCR: Relative mRNA expression of E-cad and the transcription factors OVOL1, OVOL2 and ZEB1 in the epithelial PC3-Epi cells transduced with the TSPAN8 expression lentivirus or with the empty vector control. The graph depicts the effect of TSPAN8 overexpression in the induction of EMT as shown by a decrease in E-cad and the OVOL-TFs with the concomitant increase in ZEB1.
(D) Immunoblot: Overexpression of TSPAN8 partially induces EMT in the epithelial PC3-Epi cells. TSPAN8 overexpression upregulates ZEB1 and Vimentin proteins and downregulates E-cad compared to the control epithelial PC3-Epi-EV cells. The stable mesenchymal PC3-EMT14 cells are also shown.
(E) qPCR: Analysis of TSPAN8 overexpression in the epithelial prostate cancer DU145 cells. Similar experiment as shown in (C) demonstrates the effect of TSPAN8 expression in the induction of EMT.
(F) ChIP qPCR: The graph on the left represents the input chromatin of PC3-EMT14-OVOL2 relative to empty vector (EV) control, and demonstrates that similar amounts of DNA were used. The graph on the right depicts the ChIP DNA using V5 antibody. The V5 epitope was included at the C-terminus of the expressed OVOL2. Primers used are named for their forward primer (see panel I). Results were normalized to input controls and graphs are relative to EV. Graphs show mean +/- sem; p-values are represented as *** p < 0.001.
The qPCRs and immunoblots are representative of two independent experiments with similar results.
(TIF)

Figure S3 **Mesenchymal cancer cells show decreased mouse survival in the ICI model, while not requiring MET for solid tumor formation.** Related to Figure 3.
(A) IHC: ZEB1 or E-cad staining in subcutaneous tumors. Note the high E-cad and low ZEB1 staining in the epithelial PC3-Epi compared to the mesenchymal PC3-EMT12, and PC3-EMT14. Scale bars are 50 µm.
(B) Tumor burden: Mice received subcutaneous injections and were imaged weekly for 49 days. Luciferase expression is represented as regions of interest (ROI-photons/s) as described in methods. No significant (n.s.) differences in tumor growth were observed between the mesenchymal (PC3-EMT12, and PC3-EMT14) and epithelial (PC3-Epi) cells lines.
(C) Kaplan Meier survival curves: Survival was recorded in ICI-inoculated mice with PC3-Epi, PC3-EMT12, and -EMT14.
(D) IHC: Simultaneous ZEB1 and E-cad expression in PC3-EMT12 tumors found in liver and bone from mice given ICI. Scale bar represents 100 µm.
(E) IHC: Simultaneous ZEB1 and E-cad staining of metastases sections from liver corresponding to mice ICI with PC3-Epi and PC3-EMT14 cells. Note that PC3-Epi predominately retained its epithelial phenotype, and similarly PC3-EMT14 retained its mesenchymal phenotype. Scale bars are 100 µm (black) and 20 µm (red).
The IHCs show a representative staining of one out of three sections with similar results.
(TIF)

Figure S4 **OVOL expression in mesenchymal cancer cells induces MET and forms epithelial tumors.** Related to Figure 4.
(A) IHC: E-cad and ZEB1 staining of orthotopic tumors from PC3-EMT14 expressing OVOL1 or OVOL2 and the control. Note that tumors predominantly preserved their mesenchymal (PC3-EMT14) or epithelial (PC3-EMT14-OVOL1 and OVOL2) cell origins. Scale bar represents 100 µm.
(B) IHC: E-cad, and Ki-67 staining of metastatic (peritoneum) tumor from a mouse that received an orthotopic injection with PC3-EMT14 cells. The Ki-67 staining of E-cad negative tumor cells demonstrates that these mesenchymal cells can proliferate without undergoing MET. Scale bar represents 100 µm.
The IHCs show a representative staining of one out of three sections with similar results.
(TIF)

Figure S5 **OVOL1 and OVOL2 expression correlates with hallmark genes of epithelial differentiation in 917 cancer cell lines.** Related to Figure 5.
(A) Oncomine: Gene expression analysis of the Barretina study (917 cancer cell lines) shows a significant correlation between OVOL2 and a number of epithelial associated genes including ESRP1, ESRP2, and E-cad (referred to as CDH1 on the microarray)). Within this set of genes OVOL1 (not shown) demonstrates a 0.76 correlation with OVOL2.
(B) Heat map: The top 100 genes identified in Figure 5B when comparing PC3-Epi, PC3-EMT14-OVOL1 and PC3-EMT14-OVOL2 relative to PC3-EMT14. The differential regulation (Up (red) and down (blue)) of individual genes appears to be conserved across all three epithelial cell lines.
(C) Schematic: Protein isoforms resulting from the alternative splicing of EPB41L5 induced by OVOL2. The isoform switching was identified by RNA-seq. Blue boxes represent conserved amino acid sequences, while red boxes represent unique sequences of the isoforms 1 and 4 that show upregulation in mesenchymal (EMT) or epithelial (MET) cells, respectively.
(TIF)

Table S1 **Ingenuity Pathway Analysis (IPA) of EMT cells isolated from macrophage co-cultures.** Related to Figure 1. Ingenuity Pathway analysis identified molecular functions associated with EMT like cellular movement (50 molecules), cellular and tissue development (37 molecules), and cell death (58 molecules). Function associated with cell-to-cell signaling and interaction (as occurred between cancer cells and macrophages) was also identified (49 molecules).
(DOCX)

Table S2 **Genes that positively correlate (>0.74) with OVOL1 and OVOL2 expression in 917 cancer cell lines.** Related to Figure 5. The table depicts the genes that correlate (r > 0.74) with OVOL1 and OVOL2 expression in 917 human cancer cell lines (Barretina study). Note the high correlation of E-cad, ESRP1 and ESRP2 with both OVOL1 and OVOL2 expression.
(DOCX)

Table S3 **ConceptGen analysis of common pathways associated with OVOL1 and OVOL2 induced MET signature in prostate cancer cells.** Related to Figure 5. ConceptGen analysis of the RNA-seq results showing the common pathways associated with genes that are differentially expressed in PC3-EMT14-OVOL1 and PC3-EMT14-OVOL2, relative to PC3-EMT14.
(DOCX)

Table S4 **Genes that negatively correlate (r < -0.5) with OVOL1 and OVOL2 expression in 917 cancer cell lines.** Related to Figure 5. Depicts the common genes that negatively correlate (r < -0.5) with OVOL1 and OVOL2 expression in the Barretina study (917 human cancer cell lines).
(DOCX)
