# Supplementary figures and images for: Transcription Factors OVOL1 and OVOL2 Induce the Mesenchymal to Epithelial Transition in Human Cancer
Source: PLoS One. 2013 Oct 4;8(10):e76773. doi: 10.1371/journal.pone.0076773 (PMC3790720; doi:10.1371/journal.pone.0076773)

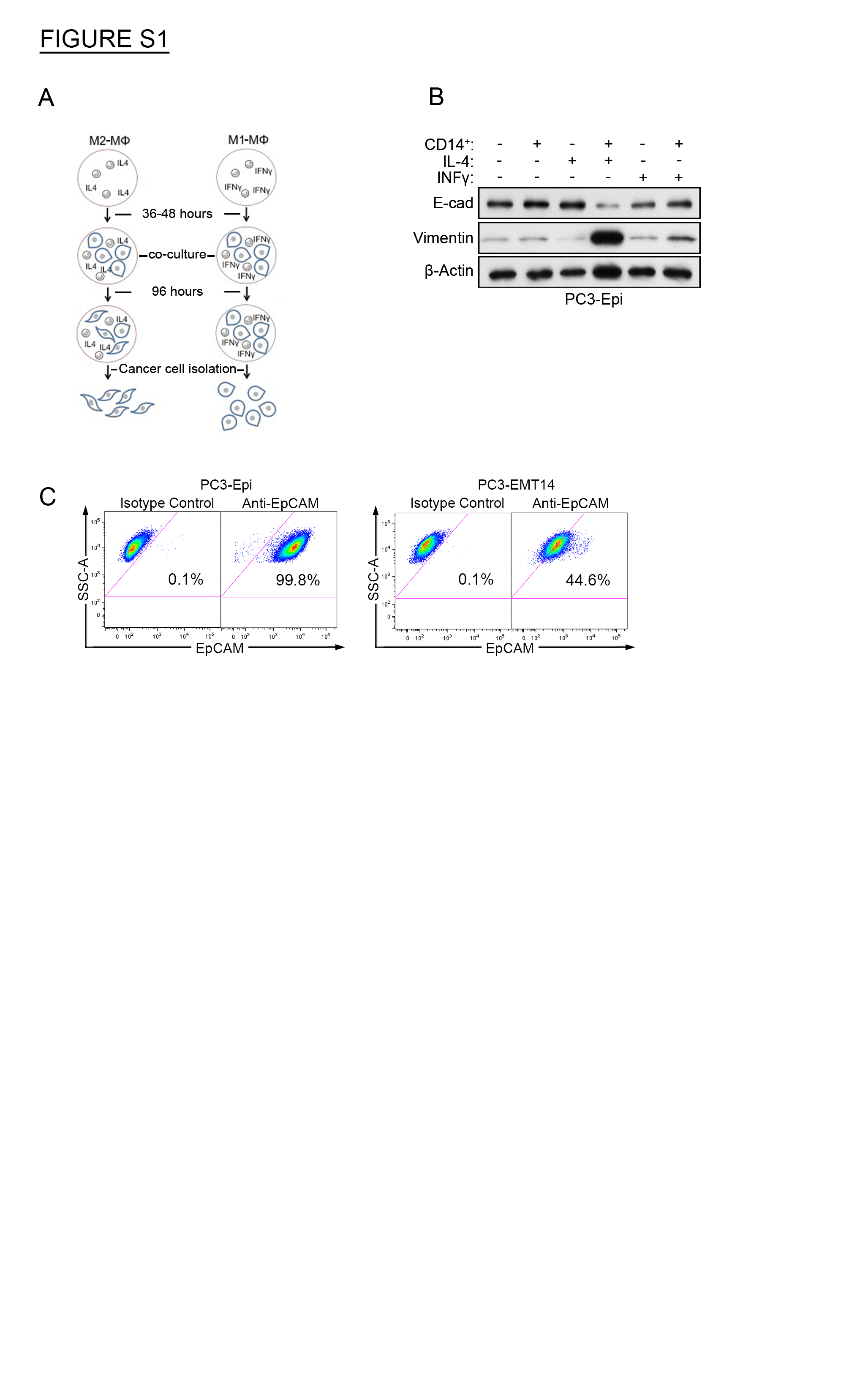

Supplement: Figure S1 — Macrophages induce EMT in epithelial prostate cancer PC3-Epi cells. Related to Figure 1. (A) Schematic: Human CD14+ blood monocytes (3x10^6^ cells) were isolated from healthy donors and induced to differentiate into M1 or M2 macrophages upon stimulation with INFγ or interleukin (IL)-4 respectively (100 ng/ml) for 36-42 hours. Next, these cells were co-cultured with 3x10^5^ highly epithelial prostate cancer cells (PC3-Epi) for 4 days. (B) Immunoblot: Shows downregulation of E-cad with the concomitant upregulation of vimentin in the cancer cell population from the co-culture with IL-4-treated CD14+ macrophages as compared to controls. The immunoblot is representative of two independent experiments with similar results. (C) Flow Cytometry: Depicts a decrease in the cell surface expression of the Epithelial Cell Adhesion Molecule (EpCAM) in the stable mesenchymal PC3-EMT14 prostate cancer cells compared to the parental epithelial PC3-Epi cells. The results are representative of two experiments. (TIF) [file pone.0076773.s001.tif]

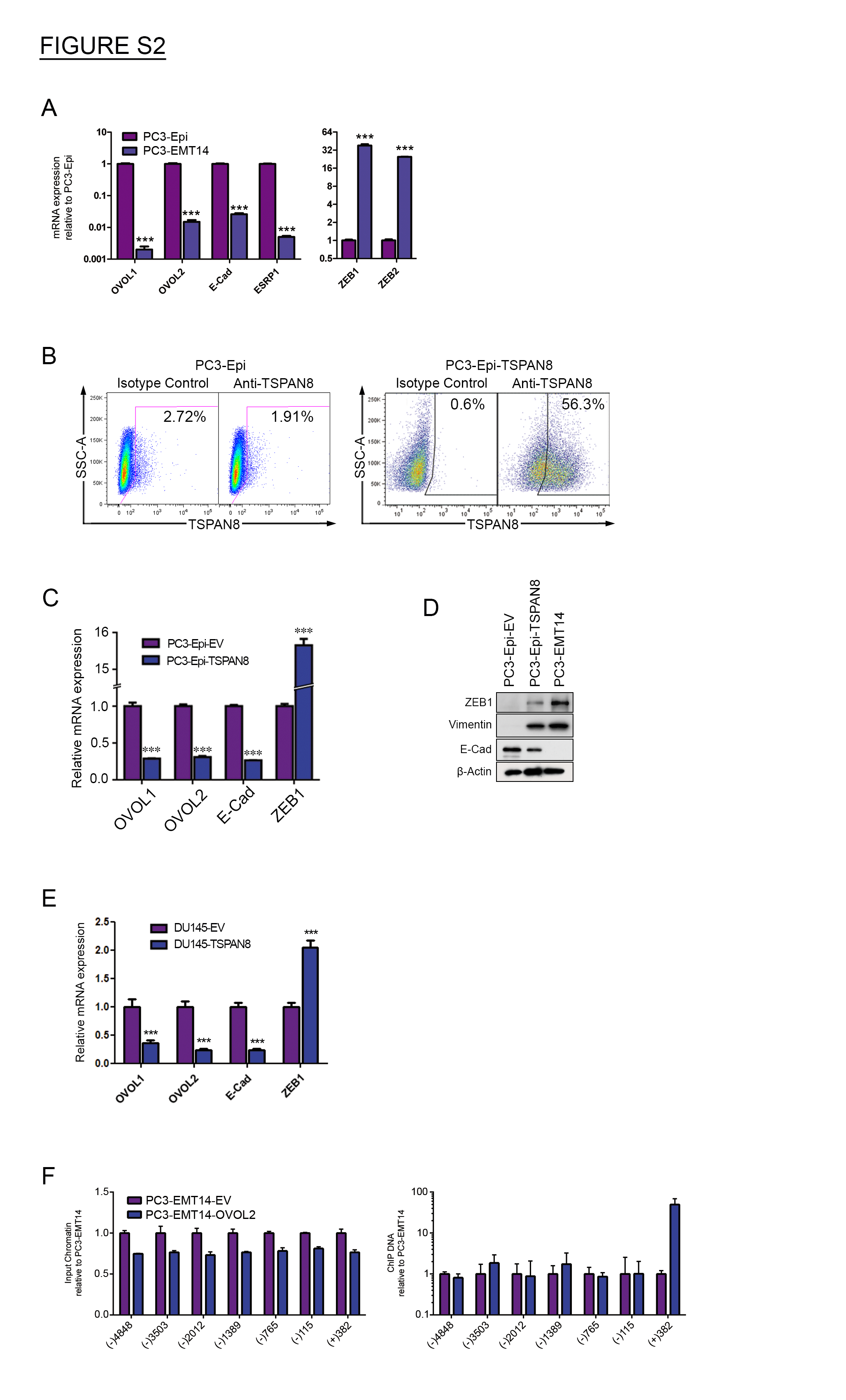

Supplement: Figure S2 — OVOL and ZEB TFs are inversely regulated in the stable epithelial (PC3-Epi) and the stable mesenchymal (PC3-EMT14) prostate cancer cells. OVOL2 binds to ZEB1 promoter. Related to Figure 2. (A) qPCR: mRNA expression in PC3-EMT14 relative to PC3-Epi prostate cancer cells. (B) Flow Cytometry: Depicts the cell surface expression of the transmembrane protein Tetraspanin-8 (TSPAN8) in the epithelial PC3-Epi cells transduced with a TSPAN8 expression lentivirus and compared to the parental PC3-Epi cells. (C) qPCR: Relative mRNA expression of E-cad and the transcription factors OVOL1, OVOL2 and ZEB1 in the epithelial PC3-Epi cells transduced with the TSPAN8 expression lentivirus or with the empty vector control. The graph depicts the effect of TSPAN8 overexpression in the induction of EMT as shown by a decrease in E-cad and the OVOL-TFs with the concomitant increase in ZEB1. (D) Immunoblot: Overexpression of TSPAN8 partially induces EMT in the epithelial PC3-Epi cells. TSPAN8 overexpression upregulates ZEB1 and Vimentin proteins and downregulates E-cad compared to the control epithelial PC3-Epi-EV cells. The stable mesenchymal PC3-EMT14 cells are also shown. (E) qPCR: Analysis of TSPAN8 overexpression in the epithelial prostate cancer DU145 cells. Similar experiment as shown in (C) demonstrates the effect of TSPAN8 expression in the induction of EMT. (F) ChIP qPCR: The graph on the left represents the input chromatin of PC3-EMT14-OVOL2 relative to empty vector (EV) control, and demonstrates that similar amounts of DNA were used. The graph on the right depicts the ChIP DNA using V5 antibody. The V5 epitope was included at the C-terminus of the expressed OVOL2. Primers used are named for their forward primer (see panel I). Results were normalized to input controls and graphs are relative to EV. Graphs show mean +/- sem; p-values are represented as *** p < 0.001. The qPCRs and immunoblots are representative of two independent experiments with similar results. (TIF) [file pone.0076773.s002.tif]

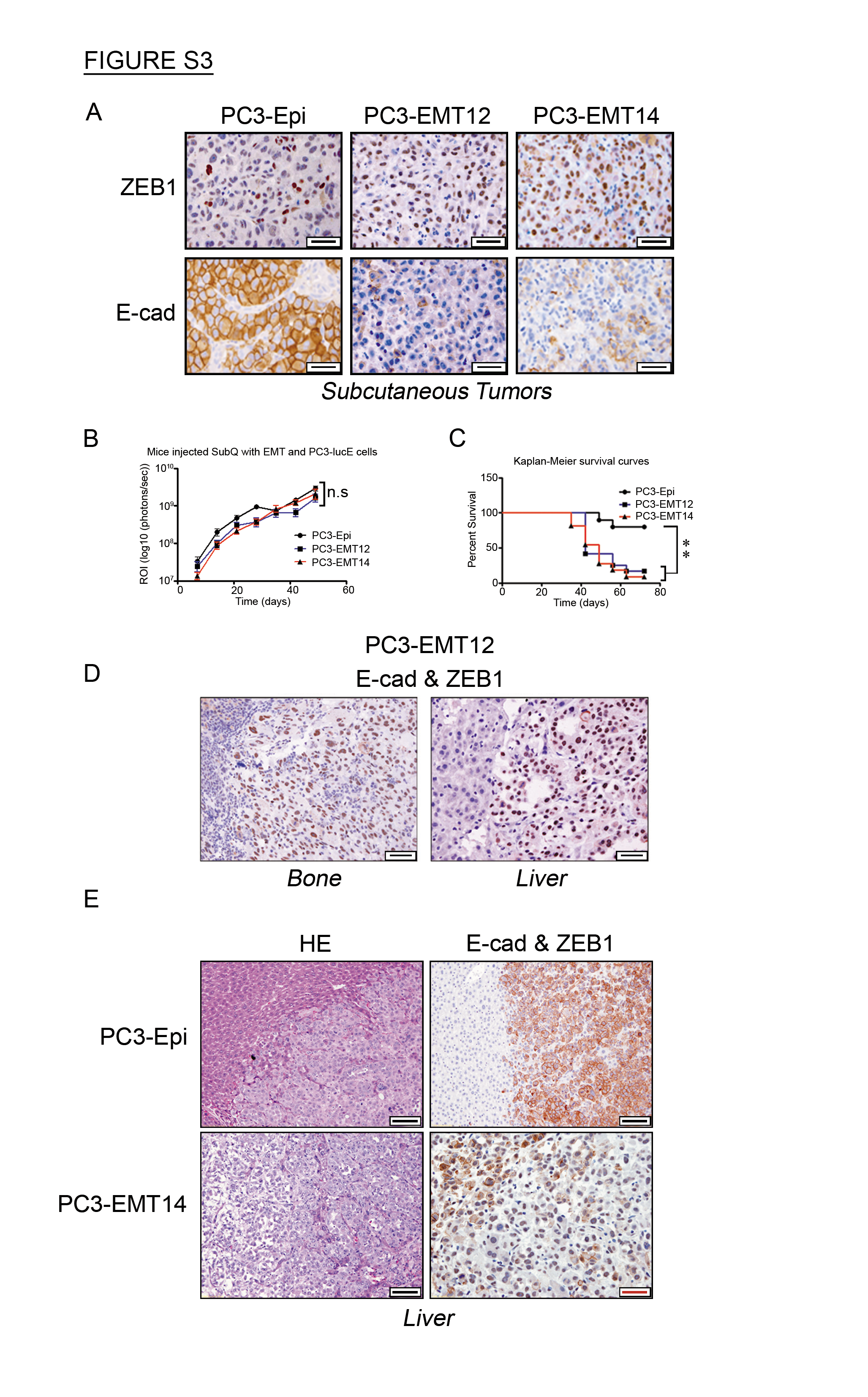

Supplement: Figure S3 — Mesenchymal cancer cells show decreased mouse survival in the ICI model, while not requiring MET for solid tumor formation. Related to Figure 3. (A) IHC: ZEB1 or E-cad staining in subcutaneous tumors. Note the high E-cad and low ZEB1 staining in the epithelial PC3-Epi compared to the mesenchymal PC3-EMT12, and PC3-EMT14. Scale bars are 50 µm. (B) Tumor burden: Mice received subcutaneous injections and were imaged weekly for 49 days. Luciferase expression is represented as regions of interest (ROI-photons/s) as described in methods. No significant (n.s.) differences in tumor growth were observed between the mesenchymal (PC3-EMT12, and PC3-EMT14) and epithelial (PC3-Epi) cells lines. (C) Kaplan Meier survival curves: Survival was recorded in ICI-inoculated mice with PC3-Epi, PC3-EMT12, and -EMT14. (D) IHC: Simultaneous ZEB1 and E-cad expression in PC3-EMT12 tumors found in liver and bone from mice given ICI. Scale bar represents 100 µm. (E) IHC: Simultaneous ZEB1 and E-cad staining of metastases sections from liver corresponding to mice ICI with PC3-Epi and PC3-EMT14 cells. Note that PC3-Epi predominately retained its epithelial phenotype, and similarly PC3-EMT14 retained its mesenchymal phenotype. Scale bars are 100 µm (black) and 20 µm (red). The IHCs show a representative staining of one out of three sections with similar results. (TIF) [file pone.0076773.s003.tif]

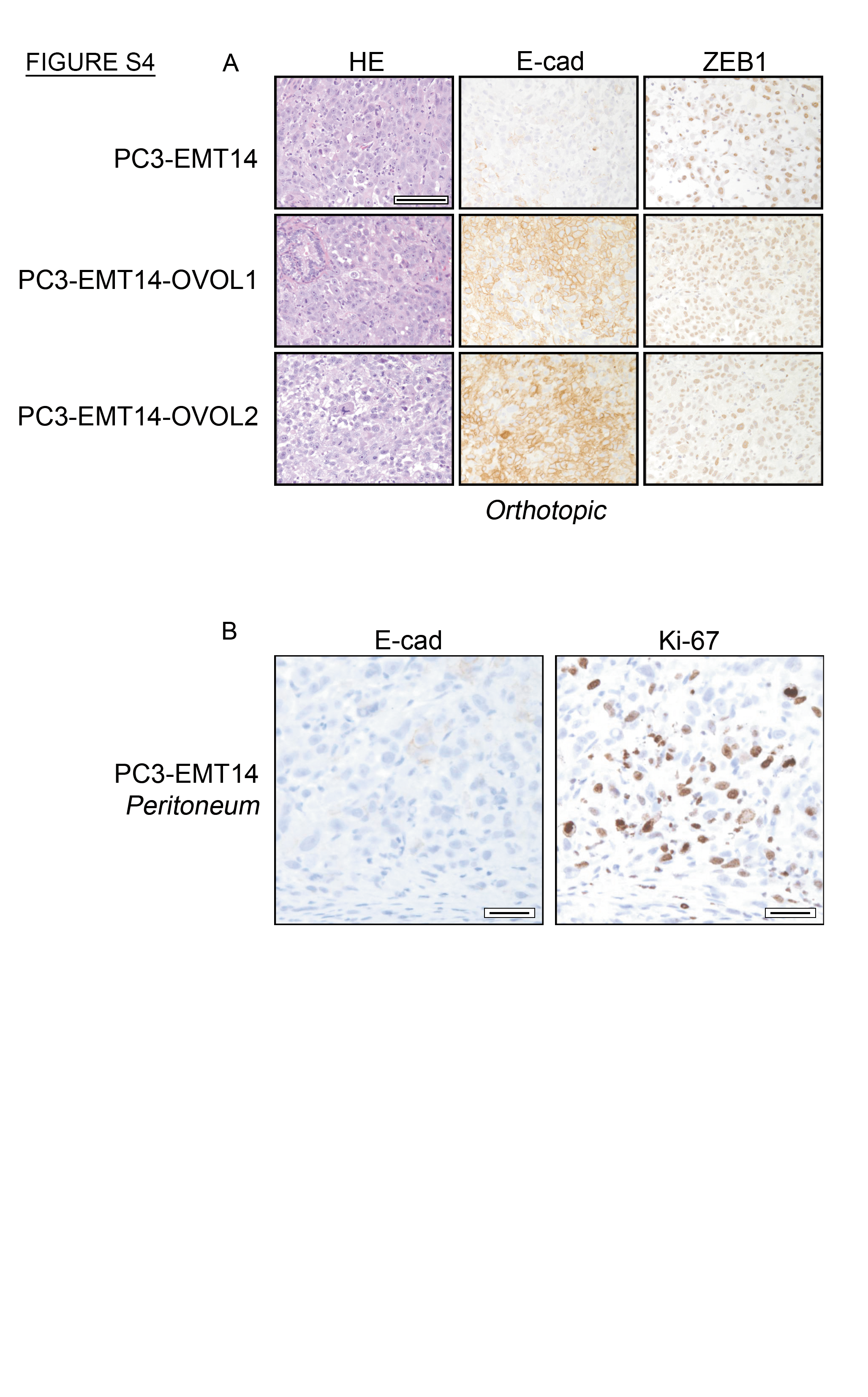

Supplement: Figure S4 — OVOL expression in mesenchymal cancer cells induces MET and forms epithelial tumors. Related to Figure 4. (A) IHC: E-cad and ZEB1 staining of orthotopic tumors from PC3-EMT14 expressing OVOL1 or OVOL2 and the control. Note that tumors predominantly preserved their mesenchymal (PC3-EMT14) or epithelial (PC3-EMT14-OVOL1 and OVOL2) cell origins. Scale bar represents 100 µm. (B) IHC: E-cad, and Ki-67 staining of metastatic (peritoneum) tumor from a mouse that received an orthotopic injection with PC3-EMT14 cells. The Ki-67 staining of E-cad negative tumor cells demonstrates that these mesenchymal cells can proliferate without undergoing MET. Scale bar represents 100 µm. The IHCs show a representative staining of one out of three sections with similar results. (TIF) [file pone.0076773.s004.tif]

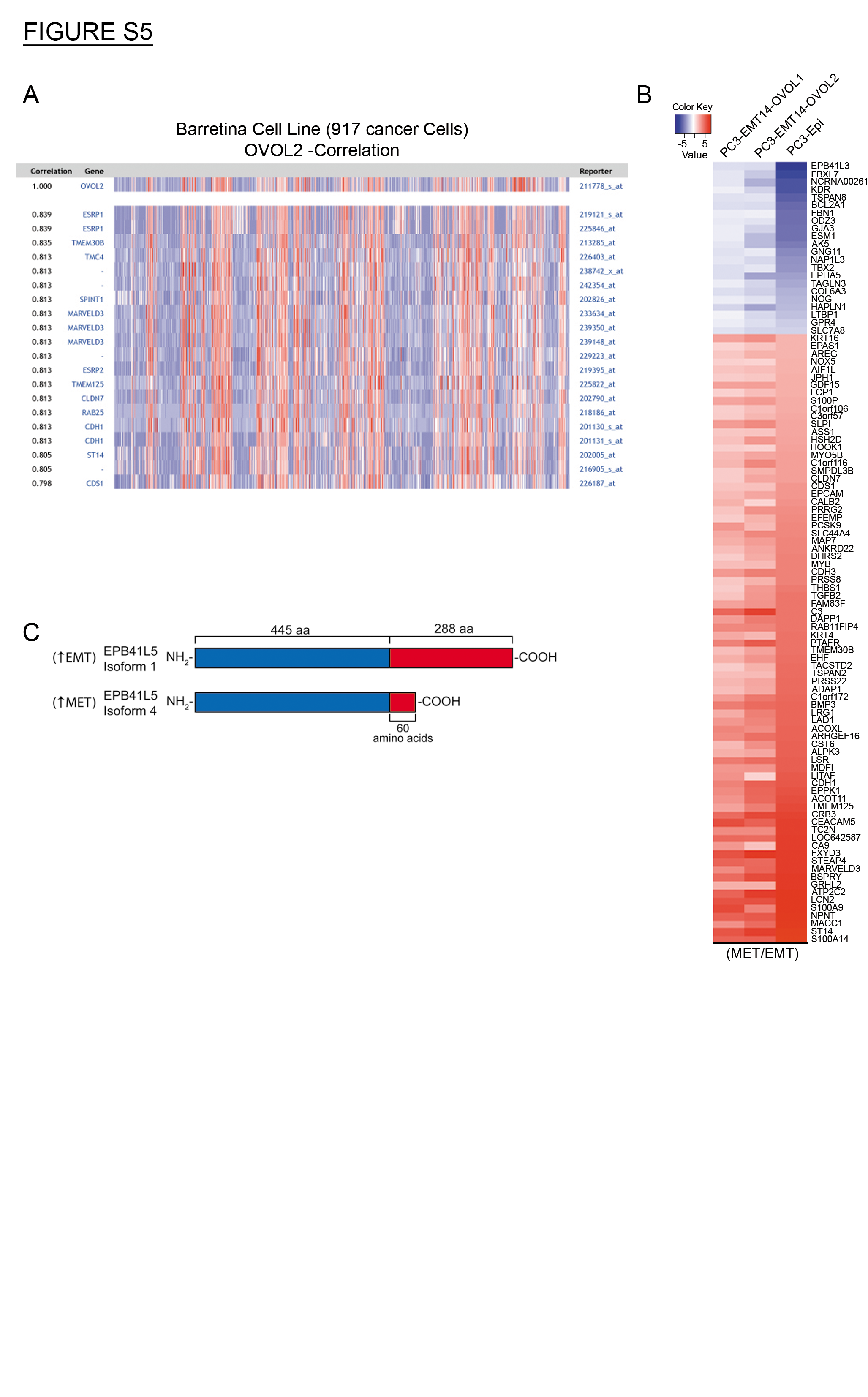

Supplement: Figure S5 — OVOL1 and OVOL2 expression correlates with hallmark genes of epithelial differentiation in 917 cancer cell lines. Related to Figure 5. (A) Oncomine: Gene expression analysis of the Barretina study (917 cancer cell lines) shows a significant correlation between OVOL2 and a number of epithelial associated genes including ESRP1, ESRP2, and E-cad (referred to as CDH1 on the microarray)). Within this set of genes OVOL1 (not shown) demonstrates a 0.76 correlation with OVOL2. (B) Heat map: The top 100 genes identified in Figure 5B when comparing PC3-Epi, PC3-EMT14-OVOL1 and PC3-EMT14-OVOL2 relative to PC3-EMT14. The differential regulation (Up (red) and down (blue)) of individual genes appears to be conserved across all three epithelial cell lines. (C) Schematic: Protein isoforms resulting from the alternative splicing of EPB41L5 induced by OVOL2. The isoform switching was identified by RNA-seq. Blue boxes represent conserved amino acid sequences, while red boxes represent unique sequences of the isoforms 1 and 4 that show upregulation in mesenchymal (EMT) or epithelial (MET) cells, respectively. (TIF) [file pone.0076773.s005.tif]
