# Supplementary material for: Transcription Factors OVOL1 and OVOL2 Induce the Mesenchymal to Epithelial Transition in Human Cancer
Source: PLoS One. 2013 Oct 4;8(10):e76773. doi: 10.1371/journal.pone.0076773 (PMC3790720; doi:10.1371/journal.pone.0076773)
Supplement: Table S1 — Ingenuity Pathway Analysis (IPA) of EMT cells isolated from macrophage co-cultures. Related to Figure 1. Ingenuity Pathway analysis identified molecular functions associated with EMT like cellular movement (50 molecules), cellular and tissue development (37 molecules), and cell death (58 molecules). Function associated with cell-to-cell signaling and interaction (as occurred between cancer cells and macrophages) was also identified (49 molecules). (DOCX) [file pone.0076773.s006.docx]

**Table S1**

| ***Ingenuity Pathway Analysis*** | | | | |
| --- | --- | --- | --- | --- |
| ***Top Networks*** | | | | |
| **ID** | **Associated Network Functions** | | | **Score** |
| 1 | Cancer, Reproductive System Disease, Cell-to-Cell Signaling and interaction | | | 39 |
| 2 | Cell Death, Tissue Development, Cellular Movement | | | 32 |
| 3 | Genetic Disorder, Skeletal and Muscular Disorders, Cancer | | | 17 |
| 4 | Cancer, Cellular Growth and Proliferation, Genetic Disorders | | | 16 |
| 5 | Cancer, Reproductive System Disease, Cellular Development | | | 16 |
| ***Molecular and Cellular Functions*** | | | | |
| **Name** | | **p-value** | **# Molecules** | |
| Cellular Movement | | 2.03E-09 – 1.86E-02 | 50 | |
| Cellular Growth and Proliferation | | 1.24E-08 – 1.69E-02 | 77 | |
| Cell-To-Cell Signaling and Interaction | | 1.11E-07 – 1.86E-02 | 49 | |
| Cell Death | | 3.55E-05 – 1.86E-02 | 58 | |
| Cellular Compromise | | 1.02E-04 – 1.86E-02 | 10 | |
| ***Physiological System Development and Function*** | | | | |
| **Name** | | **p-value** | **# Molecules** | |
| Tissue Development | | 1.11E-07 – 1.86E-02 | 37 | |
| Organismal Survival | | 1.56E-06 – 1.58E-03 | 9 | |
| Cardiovascular System Development and Function | | 3.24E-06 – 1.86E-02 | 21 | |
| Tumor Morphology | | 1.02E-04 – 1.86E-02 | 10 | |
| Hematological System Development and Function | | 1.94E-04 – 1.86E-02 | 18 | |
