# Supplementary material for: Transcription Factors OVOL1 and OVOL2 Induce the Mesenchymal to Epithelial Transition in Human Cancer
Source: PLoS One. 2013 Oct 4;8(10):e76773. doi: 10.1371/journal.pone.0076773 (PMC3790720; doi:10.1371/journal.pone.0076773)
Supplement: Table S2 — Genes that positively correlate (>0.74) with OVOL1 and OVOL2 expression in 917 cancer cell lines. Related to Figure 5. The table depicts the genes that correlate (r > 0.74) with OVOL1 and OVOL2 expression in 917 human cancer cell lines (Barretina study). Note the high correlation of E-cad, ESRP1 and ESRP2 with both OVOL1 and OVOL2 expression. (DOCX) [file pone.0076773.s007.docx]

**Table S2**

| ***Genes that Positively Correlate (>0.74) with OVOL1 and OVOL2 in 917 Cancer Cell Lines*** | | | |
| --- | --- | --- | --- |
| **Gene Name** | **Gene Symbol** | **OVOL1** | **OVOL2** |
| ovo-like 1 | OVOL1 | 0.76 | 1.00 |
| epithelial splicing regulatory protein 1 | ESRP1 | 0.76 | 0.84 |
| transmembrane protein 30B | TMEM30B | 0.76 | 0.83 |
| cadherin 1, type 1, E-cadherin (epithelial) | CDH1 | 0.76 | 0.81 |
| claudin 7 | CLDN7 | 0.76 | 0.81 |
| epithelial splicing regulatory protein 2 | ESRP2 | 0.76 | 0.81 |
| MARVEL domain containing 3 | MARVELD3 | 0.76 | 0.81 |
| RAB25, member RAS oncogene family | RAB25 | 0.76 | 0.81 |
| serine peptidase inhibitor, Kunitz type 1 | SPINT1 | 0.76 | 0.81 |
| transmembrane channel-like 4 | TMC4 | 0.76 | 0.81 |
| transmembrane protein 125 | TMEM125 | 0.76 | 0.81 |
| suppression of tumorigenicity 14 (colon carcinoma) | ST14 | 0.76 | 0.81 |
| CDP-diacylglycerol synthase (phosphatidate cytidylyltransferase) 1 | CDS1 | 0.76 | 0.80 |
| Rho GTPase activating protein 8 | ARHGAP8 | 0.76 | 0.78 |
| proline rich 5 (renal) | PRR5 | 0.76 | 0.78 |
| Cas-Br-M ecotropic retroviral transforming sequence c | CBLC | 0.79 | 0.76 |
| coiled-coil domain containing 64B | CCDC64B | 0.82 | 0.76 |
| EPH receptor A1 | EPHA1 | 0.78 | 0.76 |
| ovo-like 2 | OVOL2 | 1.00 | 0.76 |
| protease, serine, 8 | PRSS8 | 0.82 | 0.76 |
| S100 calcium binding protein A14 | S100A14 | 0.81 | 0.76 |
| epsin 3 | EPN3 | 0.76 | 0.76 |
| grainyhead-like 2 | GRHL2 | 0.76 | 0.76 |
| prominin 2 | PROM2 | 0.76 | 0.76 |
| cadherin 3, type 1, P-cadherin (placental) | CDH3 | 0.74 | 0.74 |
| interferon regulatory factor 6 | IRF6 | 0.74 | 0.74 |
