# Supplementary material for: Transcription Factors OVOL1 and OVOL2 Induce the Mesenchymal to Epithelial Transition in Human Cancer
Source: PLoS One. 2013 Oct 4;8(10):e76773. doi: 10.1371/journal.pone.0076773 (PMC3790720; doi:10.1371/journal.pone.0076773)
Supplement: Table S3 — ConceptGen analysis of common pathways associated with OVOL1 and OVOL2 induced MET signature in prostate cancer cells. Related to Figure 5. ConceptGen analysis of the RNA-seq results showing the common pathways associated with genes that are differentially expressed in PC3-EMT14-OVOL1 and PC3-EMT14-OVOL2, relative to PC3-EMT14. (DOCX) [file pone.0076773.s008.docx]

**Table S3**

| ***ConceptGen Analysis of Common Pathways Associated with OVOL1 and OVOL2 Induced MET in PCa Cells*** | | | |
| --- | --- | --- | --- |
| **Concept Name** | **# of Genes** | **P-Value** | **NCBI Gene ID** |
| Genes, Dominant | 9 | 4.80E-08 | 6785,255738,2700,30061,9132,3664,7045,2707,2200 |
| Epithelial Cells | 9 | 9.15E-08 | 7042,10207,374,92359,1366,6591,4950,3576,999 |
| Immunohistochemistry | 9 | 8.80E-07 | 6781,1001,10630,5156,1366,3791,768,794,999, |
| Calcium | 11 | 3.88E-06 | 3936,6781,6286,5321,7421,7852,7135,6280,6857,7057,2200 |
| Gene Expression Regulation, Neoplastic | 10 | 4.71E-06 | 3481,6286,5156,5655,2034,9518,7852,768,238,999 |
| Tumor Markers, Biological | 8 | 7.02E-06 | 10630,6317,3485,1048,57016,768,794,999 |
| Endothelium, Vascular | 8 | 8.03E-06 | 7075,3791,7852,3690,4950,11082,7057,3576 |
| Epithelium | 5 | 1.18E-05 | 26298,6591,4950,6590,999 |
| Membrane Proteins | 17 | 1.26E-05 | 10207,3714,2044,22871,6785,2043,5742,23209,6857,79400,27134,5349,4050,23136,1366,58191,4950 |
| EGF-like domain | 12 | 1.26E-05 | 3714,374,7075,5742,10178,84628,4753,3690,255743,4052,7057,2200 |
| calcium ion binding | 33 | 1.30E-05 | 1004,1001,6286,50853,255738,4753,6280,5639,23208,54836,4052,4325,2202,411,2200,4324,3714,84440,954,5321,9914,255743,9066,794,23624,6857,3936,57402,79400,11240,50859,7057,999 |
| extracellular region part | 32 | 1.67E-05 | 1293,9241,255738,51599,3898,6280,727,6414,2246,4050,3485,84628,4052,4325,2202,7045,3576,4324,2200,6781,7483,651,1404,255743,5652,23452,374,6367,80326,9518,58191,50859 |
| Insulin-Like Growth Factor Binding Protein 4 | 4 | 2.03E-05 | 3481,3485,3488,3487 |
| Insulin-Like Growth Factor Binding Protein 1 | 4 | 4.31E-05 | 3481,3485,3488,3487 |
| Insulin-Like Growth Factor Binding Protein 5 | 4 | 4.31E-05 | 3481,3485,3488,3487 |
| Mesoderm | 5 | 4.76E-05 | 7042,9241,255743,6591,999 |
| Transforming Growth Factor beta | 7 | 5.04E-05 | 7042,9241,651,9518,4052,7045,7057 |
| Cell Movement | 8 | 5.72E-05 | 2886,6367,7852,3791,58191,3690,7057,999, |
| Somatomedins | 4 | 7.82E-05 | 3481,3485,3488,3487, |
| Thyroglobulin type-1 repeat | 5 | 8.26E-05 | 4070,3485,3488,3487,50859, |
| Insulin-Like Growth Factor Binding Protein 3 | 4 | 1.01E-04 | 3481,3485,3488,3487, |
| Platelet Aggregation | 5 | 1.17E-04 | 10630,2043,5742,3690,7057, |
| Insulin-Like Growth Factor Binding Protein 2 | 4 | 1.28E-04 | 3481,3485,3488,3487, |
| Receptor Protein-Tyrosine Kinases | 7 | 1.39E-04 | 2044,2246,2045,7075,2043,3791,238, |
| Vascular Endothelial Growth Factor A | 5 | 1.99E-04 | 2034,3791,7057,11082,3576, |
