# Supplementary material for: Transcription Factors OVOL1 and OVOL2 Induce the Mesenchymal to Epithelial Transition in Human Cancer
Source: PLoS One. 2013 Oct 4;8(10):e76773. doi: 10.1371/journal.pone.0076773 (PMC3790720; doi:10.1371/journal.pone.0076773)
Supplement: Table S4 — Genes that negatively correlate (r < -0.5) with OVOL1 and OVOL2 expression in 917 cancer cell lines. Related to Figure 5. Depicts the common genes that negatively correlate (r < -0.5) with OVOL1 and OVOL2 expression in the Barretina study (917 human cancer cell lines). (DOCX) [file pone.0076773.s009.docx]

**Table S4**

| ***Genes that Negatively Correlate (r < -0.5) with OVOL1 and OVOL2 in 917 Cancer Cell Lines*** | | | |
| --- | --- | --- | --- |
| **Reporter** | **Gene ID** | **OVOL1** | **OVOL2** |
| 225793_at | LIX1L | -0.79917 | -0.7698 |
| 212758_s_at | ZEB1 | -0.73634 | -0.70673 |
| 223024_at | AP1M1 | -0.62932 | -0.70636 |
| 201426_s_at | VIM | -0.67534 | -0.70259 |
| 36030_at | IFFO1 | -0.57003 | -0.6688 |
| 203729_at | EMP3 | -0.52823 | -0.65396 |
| 227295_at | IKBIP | -0.58129 | -0.65063 |
| 225045_at | CCDC88A | -0.7307 | -0.64732 |
| 230264_s_at | AP1S2 | -0.66131 | -0.62491 |
| 220750_s_at | LEPRE1 | -0.62307 | -0.62286 |
| 203603_s_at | ZEB2 | -0.52804 | -0.62213 |
| 202664_at | WIPF1 | -0.56448 | -0.61936 |
| 213262_at | SACS | -0.62196 | -0.60421 |
| 229336_at | ST3GAL2 | -0.50779 | -0.59122 |
| 212746_s_at | CEP170 | -0.66525 | -0.58372 |
| 238418_at | SLC35B4 | -0.60153 | -0.58064 |
| 205407_at | RECK | -0.53345 | -0.56049 |
| 200600_at | MSN | -0.50095 | -0.54683 |
| 226771_at | ATP8B2 | -0.58662 | -0.54273 |
| 209447_at | SYNE1 | -0.5237 | -0.52556 |
| 213434_at | STX2 | -0.56161 | -0.52426 |
| 210151_s_at | DYRK3 | -0.52586 | -0.50043 |
